# A comprehensive analysis of the emerging modern trends in research on photovoltaic systems and desalination in the era of artificial intelligence and machine learning

**DOI:** 10.1016/j.heliyon.2024.e25407

**Published:** 2024-02-01

**Authors:** Laxmikant D. Jathar, Keval Nikam, Umesh V. Awasarmol, Raviraj Gurav, Jitendra D. Patil, Kiran Shahapurkar, Manzoore Elahi M. Soudagar, T. M. Yunus Khan, M.A. Kalam, Anna Hnydiuk-Stefan, Ali Etem Gürel, Anh Tuan Hoang, Ümit Ağbulut

**Affiliations:** a Department of Mechanical Engineering, Army Institute of Technology Pune, Maharashtra, 411015, India; b Department of Mechanical Engineering, Dr. D. Y. Patil Institute of Engineering, Management and Research, Akurdi, Pune, 411044, India; c Department of Mechanical Engineering, School of Mechanical, Chemical and Materials Engineering, Adama Science and Technology University, Adama, 1888, Ethiopia; d Faculty of Mechanical Engineering, Opole University of Technology, 45-758 Opole, Poland; e Department of Mechanical Engineering, Graphic Era (Deemed to Be University), Dehradun, Uttarakhand, 248002, India; f Department of Mechanical Engineering, College of Engineering, King Khalid University, Abha, 61421, Saudi Arabia; g School of Civil and Environmental Engineering, FEIT, University of Technology Sydney, Sydney, NSW, 2007, Australia; h Faculty of Production Engineering and Logistics, Opole University of Technology, 45-758 Opole, Poland; i Department of Electricity and Energy, Düzce Vocational School, Düzce University, 81010, Düzce, Turkiye; j Faculty of Automotive Engineering, Dong A University, Danang, Viet Nam; k Department of Mechanical Engineering, Mechanical Engineering Faculty, Yildiz Technical University, İstanbul, Turkiye

**Keywords:** Artificial intelligence, Machine learning, Photovoltaic, Desalination

## Abstract

Integration of photovoltaic (PV) systems, desalination technologies, and Artificial Intelligence (AI) combined with Machine Learning (ML) has introduced a new era of remarkable research and innovation. This review article thoroughly examines the recent advancements in the field, focusing on the interplay between PV systems and water desalination within the framework of AI and ML applications, along with it analyses current research to identify significant patterns, obstacles, and prospects in this interdisciplinary field. Furthermore, review examines the incorporation of AI and ML methods in improving the performance of PV systems. This includes raising their efficiency, implementing predictive maintenance strategies, and enabling real-time monitoring. It also explores the transformative influence of intelligent algorithms on desalination techniques, specifically addressing concerns pertaining to energy usage, scalability, and environmental sustainability. This article provides a thorough analysis of the current literature, identifying areas where research is lacking and suggesting potential future avenues for investigation. These advancements have resulted in increased efficiency, decreased expenses, and improved sustainability of PV system. By utilizing artificial intelligence technologies, freshwater productivity can increase by 10 % and efficiency. This review offers significant and informative perspectives for researchers, engineers, and policymakers involved in renewable energy and water technology. It sheds light on the latest advancements in photovoltaic systems and desalination, which are facilitated by AI and ML. The review aims to guide towards a more sustainable and technologically advanced future.

## Nomenclature

AI: Artificial Intelligence
ML: Machine Learning
PV: Photovoltaic
ANN: Artificial Neural Network
MPPT: Maximum power point tracking
DL: Deep Learning
SS: Solar Stills
GA: Genetic Algorithm
SDM: Single Diode Model
DDM: Double Diode Model
TDM: Triple Diode Model
NRM: Newton Raphson Method
NLS: Non-Linear Least Square
HS: Harmony Search Algorithm
BMO: Bird Mating Optimizer
GOA: Grasshopper Optimization Algorithm (GOA)
SMA: Slime Mould Algorithm
FADE: Fuzzy Adaptive Differential Evolution Algorithm
MSFS: Modified-Stochastic-Fractal-Search Algorithm
PGJAYA: Performance-Guided JAYA
COA: Coyote Optimization Algorithm
SDO: Supply-Demand-Based Optimization
LCC: Life Cycle Costing
T-S: Takagi-Sugano Fuzzy Modelling
FLC: Fuzzy Logic Controller
IPSO: Improved Particle Swarm Optimization
SMC: Sliding Mode Controller
OIACO-BPNN: Improved Ant-Colony-Optimization Algorithm-Trained BP Neural Network
PI: Proportional Integral
SVM: Support Vector Machines
GEP: Gene Expression Programming
BPNN: Back Propagation Neural Network
RBL-ELM: Extreme Learning Machine-Radial Basis Function Networks
PNN: Probabilistic Neural Network
MLR: Multilinear Regression
DO: Dissolved Oxygen
BOD: Biochemical Oxygen Demand
FA-RVFL: Firefly algorithm Random vector functional link network
LSSVM: Least Squares Support Vector Machine
FF: Feed Forward
GMPP: global maxima peak power
POD: phase opposite disposition
WQI: Water quality index
PI: Parameter Identification
ANFIS: Adaptive neuro fuzzy inference system
AIS: Artificial Immune System
PSO: Particle Swarm Optimization
CSO: Cuckoo Search Optimizer
ICSO: Improved Cuckoo Search Optimizer
MCSO: Modified Cuckoo Search Optimizer
SDOA: Supply-Demand-Based Optimization Algorithm
TFWO: Turbulent Flow of Water-Based Optimization
HHO: Harris Hawk Optimization
GTO: Gorilla Troops Optimization
FBIA: Forensic-Based Investigation Algorithm
CLPSO: Closed-loop Particle Swarm Optimization
EHO: Elephant Herd-Optimization
IBO: Improved Bonobo Optimizer
GWO: Grey Wolf Optimization
ACS: Adaptive Compass Search
GAMNU: Genetic Algorithm Based on Non-Uniform Mutation
NGO: Northern Goshawk Optimization
EGBO: Enhanced Gradient Based Optimizer
MPA: Marine Predators Algorithm
RMSE: Root-Mean-Square Deviation
P&O: Perturb and observe
MPP: Maximum Power Point
EA: Evolutionary Algorithms
VPSO-LF: Velocity of PSO-based Levy Flight
ACO: Ant Colony Optimization
SRM: Switched Reluctance Motor
RBF: Radial Basis Function
k-NN: k-Nearest Neighbours
WT: Wavelet Transform
LAPART: Laterally Primed Adaptive Resonance Theory
M-SVM: Multi-Class Support Vector Machine
RF: Random Forest
TSS: Total Dissolved Solids
SWR: Stepwise Regression (SWR)
ICA: Imperialist Competition Algorithm
MARS: Multivariate Adaptive Regression Analysis
MT: M5Model Tree approaches
TLABC: teaching learning-based and artificial bee colony
MLDCLI: Maximum Power Point Tracking and Load Current Injection
MCPWM: multiple of carrier pulse width modulation

## 1 Introduction

Recent advancements in technology have not only provided wealth to the world but also considerably improved the amount of energy that is used globally. The depletion of finite fossil fuel resources and the imminent danger of climate change have prompted numerous nations to explore the feasibility of harnessing renewable energy sources. Solar energy is now the green energy source that is expanding at the fastest rate due to the fact that it is convenient, simple to operate, safe, and reliable [1]. However, one of the most significant challenges that humankind is now contending with is climate change [2]. It is now very necessary for the government and the many companies that provide energy solutions to come up with a renewable energy system that can be maintained throughout time in order to meet the challenges that they face.

Artificial intelligence (AI) and machine learning (ML) have developed important technological solutions in recent years as the energy sector continues to search for new ways to meet the ever-increasing need for energy that is reliable, affordable, and environmentally friendly [3]. By providing them with a comprehensive set of training instructions, it instructs computer systems to learn, reason, and make judgments in a manner that is analogous to that of humans [4]. These highly developed technologies have the ability to investigate the past, improve the present, and make accurate forecasts about the future. This indicates that AI and ML have the ability to come up with solutions to the majority of the difficulties that currently exist. Despite this, there are still a few difficulties that need to be solved, and these are challenges that can be overcome with the assistance of AI and ML [5]. Renewable energy has an extensive range of positive effects. To manage the grid in a manner that is more effective; the renewable energy sector will need to improve its forecasting capabilities as well as its scheduling of electricity supplies. There are effective technologies that can predict the weather conditions, but there are likely to be unexpected shifts in the environment that can disrupt the flow of electricity. The distribution network for renewable energy is susceptible to these kinds of weaknesses [6]. It needs to be refined to a sufficient degree so that it can accommodate unforeseen changes. Second, although there have been some recent improvements in energy storage technology, the technology is still in its infant stages and requires extensive testing. There is no question that the demand for renewable energy will raise within the near upcoming years. Because of this, it is becoming increasingly important to make investments in developing technologies like AI, ML and IoT in order to boost productivity and make up for shortages [7]. In accordance with research that was just published by DNV GL, artificial intelligence (AI) will progressively mechanize processes in the subsequent centuries in the solar and wind sectors, which will enhance efficiencies all across renewable energy sector.

Research in the domains of photovoltaic (PV) systems and desalination is undergoing constant development, propelled by technological breakthroughs and the imperative for sustainable energy solutions. The contemporary research on PV systems and desalination encompasses various emerging trends. These trends frequently involve the integration of converters, Maximum Power Point Tracking (MPPT) techniques, reconfiguration strategies, concentrators, collectors, solar still designs, and the integration of AI and ML applications. Researchers are currently engaged in the development of innovative solar cell technologies, including perovskite solar cells and multi-junction solar cells, with the aim of improving conversion efficiency and mitigating manufacturing expenses [8]. Bifacial solar panels possess the capability to harness sunlight from both the front and back surfaces, hence augmenting their overall efficiency. The research is centred on the optimization of the design and integration of these entities within diverse contexts [9]. The investigation of MPPT algorithms and converters is now underway to enhance the effectiveness of energy extraction from photovoltaic (PV) panels. This research primarily focuses on adjusting the operational theme of the system in response to changing weather conditions, aiming to optimize energy extraction efficiency [10]. The implementation of smart grid technology facilitates the establishment of bidirectional communication channels between utility providers and consumers, hence enhancing the management of energy distribution, consumption, and storage [11].

While in case of solar desalination the research primarily centres around enhancing the designs of solar stills, integrating modern materials, and optimising the geometry to achieve greater efficiency in water desalination procedures [12,13]. Concentrated solar desalination systems employ reflective surfaces such as mirrors or lenses to concentrate sunlight, hence elevating the temperature and enhancing the effectiveness of the desalination process. Current research endeavours are focused on optimising concentrator designs and enhancing energy efficiency [14,15]. The combination of solar desalination and other desalination techniques, such as reverse osmosis, to form hybrid systems that leverage the respective advantages of each method, resulting in enhanced efficiency and reduced expenses [16,17]. AI and ML algorithms are utilized in the optimization of desalination processes through the analysis of real-time data. These algorithms enable the prediction of system behaviour and facilitate the adjustment of operational parameters to achieve maximum efficiency and minimise energy usage [18,19]. The research primarily centres around zero liquid discharge (ZLD) systems, which aim to achieve complete purification and recycling of wastewater, hence minimizing its environmental footprint. AI and ML techniques are utilized in the practical implementation of real-time monitoring and control systems for ZLD operations [20]. In brief, contemporary investigations in the field of photovoltaic systems and desalination exhibit notable progressions in solar cell technologies, power electronics and converters with enhanced efficiency, inventive approaches to desalination, incorporation of AI and ML for optimization and predictive maintenance, and a concerted emphasis on sustainability and mitigating environmental impact. These trends collectively lead to the advancement of more effectual, economically viable, and ecologically sustainable technologies for energy generation and water desalination.

The integration of AI and ML technology has led to substantial improvements in the domains dealing with PV and desalination in latest years. The emergence of these contemporary trends has fundamentally transformed the field of research and development in sustainable energy and freshwater production. Nevertheless, in the middle of the rapid increase of scientific studies, there is a crucial requirement for a thorough analysis that combines the various aspects of these trends. The existing literature fails to offer a thorough examination and integration of the various approaches, uses, and results arising from the combination of PV systems, desalination processes, and AI/ML technologies. The lack of a comprehensive knowledge base impedes the capacity of researchers, policymakers, and industry professionals to fully understand the overall influence and possibilities of these advancements. Moreover, the lack of a cohesive framework obstructs the ability to pinpoint deficiencies, difficulties, and prospects in this interdisciplinary field. It is imperative to address this discrepancy for multiple reasons. Firstly, comprehending the interdependent connection between solar systems, desalination processes, and AI/ML algorithms can result in the creation of more effective, environmentally friendly, and economically feasible solutions for producing clean energy and generating freshwater. Furthermore, conducting such an analysis is crucial in order to direct future research efforts, allowing researchers to concentrate on the most advantageous areas of investigation. Furthermore, policymakers and industry stakeholders necessitate a comprehensive comprehension of these patterns in order to develop well-informed policies and investment plans that promote the advancement of eco-friendly energy and water technology.

Hence, the objective of this study is to do a meticulous and all-encompassing examination of the latest advancements in research on photovoltaic systems and desalination in the era of AI and ML. This review strives to provide a exhaustive and detailed analysis of the present state of knowledge by synthesizing existing research, evaluating approaches, and dissecting outcomes. By doing this research, we will be able to identify areas where our understanding is lacking, technological obstacles, and ethical concerns. This will enable us to determine future research paths and strategic actions in the fields of sustainable energy and water management. The current study presents the following general framework: Necessity and importance of AI-ML in PV system and solar desalination is represented in Section 1; Conventional algorithm weaknesses and AI solutions are elaborated in Section 2; Utilization of Artificial Intelligence in PV- Systems discussed in Section 3; Section 4 elaborates about the utilization of Artificial Intelligence in a desalination process operated by renewable energy; Section 5, 6 discusses about challenges in AI-ML implementation and recommendations from the results of current review article; Finally, Section 7, 8 represents conclusions and future directions.

### 1.1 Significant contribution of this work and the purpose of choosing the PV and solar still

The selection of photovoltaic (PV) systems and solar stills for investigation can serve multiple purposes, encompassing a range of scientific, environmental, and practical factors. There are several justifications for selecting these technologies for investigation. Photovoltaic (PV) systems and solar stills effectively capture and utilize solar energy [12,21]. The examination of these technologies facilitates comprehension of the effective methods for harnessing solar energy across diverse applications, hence diminishing the dependence on non-renewable energy sources such as fossil fuels [22]. Fossil fuels provide a substantial contribution to the degradation of the environment and the alteration of global climate patterns. Photovoltaic (PV) systems and solar stills offer environmentally friendly energy solutions by generating electricity without emitting greenhouse gasses or any other detrimental substances. The examination of these technologies facilitates the advancement of ecologically sustainable alternatives and the mitigation of climate change effects [23]. Photovoltaic (PV) systems and solar stills play a crucial role in delivering electricity and potable water to underserved regions that lack traditional infrastructure. The study of these technologies contributes to the development of efficient systems specifically designed for these surroundings, thereby enhancing living circumstances and promoting sustainable development [24]. The field of solar energy is always evolving. Research and investigation in the field of photovoltaic (PV) systems and solar stills play a significant role in driving technical progress, resulting in the development of more efficient, economically viable, and easily expandable solutions. Enhancing comprehension of the fundamental principles and advancing the development of these technologies has the potential to expedite their widespread implementation on a worldwide scale [25]. The examination of the economic feasibility of solar technologies becomes increasingly imperative as their efficiency and cost-effectiveness improve. The examination of economic factors, such as return on investments, payback periods, and subsidies, enables decision-makers and enterprises to make well-informed decisions pertaining to the adoption of solar power and desalination systems [26,27]. In brief, the selection of photovoltaic (PV) systems and solar stills for research purposes comprises a wide-ranging objective that includes the preservation of the environment, the promotion of sustainable development, the advancement of technology, and the enhancement of the quality of life for global communities.

### 1.2 Necessity of AI-ML in photovoltaic

In today's world, solar power generation accounts for a considerable quantity of all renewable energy use. In addition, it is anticipated that the global solar photovoltaic (PV) capacity will expand from 593.9 GW in 2019 to 1582.9 GW in 2030, and this increase will be due to capacity expansions made by India, Germany, the United States, and Japan [5]. Nevertheless, the implementation of PV systems still entails high prices and concerns with efficacy which need to be rectified [28]. Continued attempts are being undertaken in order to reduce the costs associated with installing photovoltaic (PV) systems while also improving their efficiency, making their installation simpler, and improving their ability to couple to power grids.

In order to solve these issues, AI- ML algorithms have emerged as an alternative to more conventional approaches to the problem of providing solutions that increase the performance of photovoltaic (PV) systems. In particular, advancements in deep learning (DL) for PV systems over the past 5 years have accelerated study in these areas, with the result being more robust models for analysing structured data of all types. This was made possible by the advances in DL that occurred during the last five years. As a result, it is absolutely necessary to conduct research into novel approaches that solve the issues plaguing PV systems by making use of the most advanced models now available in the field of AI-ML.

### 1.3 Necessity of AI-ML in solar desalination (solar still)

**Fig. 1:**
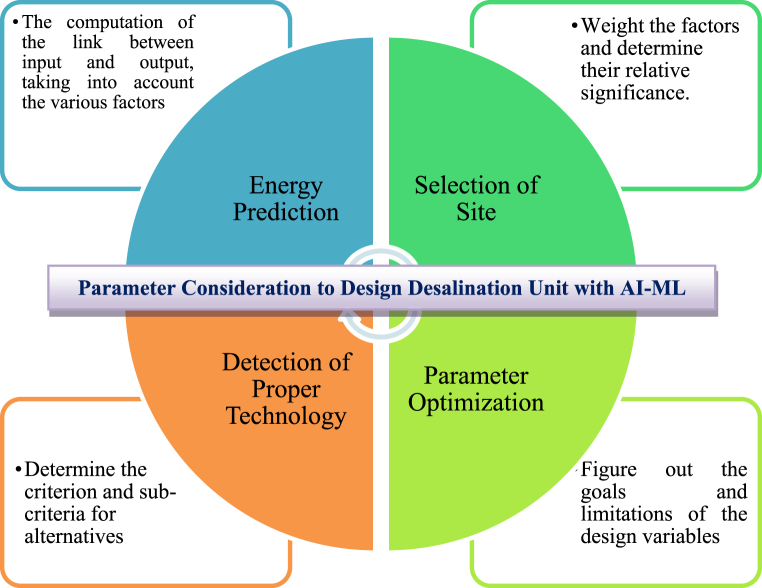
**Parameters in Desalination Systems where AI-ML is applied** [18] **(Adapted with permission from ELSEV. B.V. with LIC. No 5517441249037)**.

The availability of water that is safe to drink is one of the most valuable resources necessary to maintain human life on earth [29,30]. In a world, about 97 % of the water sources are salty, there are approximately 800 million individuals who do not have source of clean drinking water [31]. In addition to this, it is predicted that up to year 2050, about half of the water on the planet will have been utilized [18,32]. There are a variety of approaches to water desalination that can be applied to tackle this challenge successfully. Solar stills (SSs) are the most prevalent method for desalinating water due to the ease with which it can be constructed, and the low cost at which it can provide clean water [33]. AI-ML has been implemented in a variety of engineering specializations, comprising as purification and water management applications, and it can serves an essential part in exploiting the efficiency with which unavoidable variations in process conditions are optimized [34]. The modelling proficiencies of AI approaches are extremely helpful in water filtration and sewage treatment methods. This is due to the fact that the mechanization of these provisions resulted in simple and inexpensive actions, as well as a considerable drop in the possibility of manual mistakes. Fig. 1 represents the parameters in desalination systems where AI-ML is to be applied.

### 1.4 Importance of AI-ML in PV and solar still

AI and ML have emerged as pivotal components within the domain of solar PV and solar stills because of their distinct characteristics and inherent value. These technologies offer a multitude of factors that contribute to their significance in this specific field [35]. PV systems and solar stills are intricate systems that involve multiple variables, including weather conditions, sunshine intensity, temperature, and system configurations [36,37]. AI and ML algorithms provide the capability to effectively manage intricate scenarios and extract patterns from extensive information, hence facilitating accurate forecasts and optimisations. AI and ML facilitate the implementation of data-centric approaches to decision-making. Through the examination of historical data, these technologies have the capability to offer valuable insights pertaining to energy production, efficiency, and the behaviour of systems. The provided knowledge holds significant value in terms of enhancing the design and functionality of PV systems and solar stills [38]. Machine learning algorithms have the capability to forecast future solar energy generation by utilizing historical data and the prevailing environmental circumstances. Accurate forecasting is of paramount importance for utilities and grid operators in order to efficiently manage the balance between energy supply and demand [39]. Artificial intelligence demonstrates exceptional proficiency in identifying complex patterns within datasets. In the context of solar systems, these patterns may be associated with variations in meteorological conditions, fluctuations in energy production, or instances of system malfunctions. Machine learning algorithms have the capability to detect and analyse these patterns, hence facilitating comprehension and regulation of the system [40]. Machine learning models have the capability to be customised to suit certain solar configurations. These systems have the ability to adjust and conform to various climatic circumstances and system configurations, hence offering tailored solutions for a wide range of PV and solar still applications [41].

## 2 Conventional algorithm weaknesses and AI solutions

Over the course of the past few decades, artificial intelligence systems have established their feasibility as viable alternatives to traditional methods of information processing [42]. Applications of AI modelling revealed that they are proficient of tackling a extensive variability of problems with any level of complexity and difficulty [43,44]. When compared to more traditional methods, this is the most notable quality that AI technologies possess. In contrast, for the latter scenario, to minimise the complication of the system under study, it is required to assume a few things [45]. For instance, the formulation of predictor variables should be created for each potential combination of independent factors in order to improve and more accurately forecast the removal of a specific pollutant during the treatment process. Specifically, this should involve changing just one variable at a time while maintaining the status quo for the remaining variables [46]. Due to absence of comprehension regarding the complexity of the system, these assumptions can unexpectedly lead to specific differences between theoretical models and experimental data, which can hinder the accurateness of the model [47,48]. One more characteristic of AI tools is that they do not necessitate an in-depth understanding of the phenomenon or the process that is being investigated. To be more specific, it is not necessary to have numerical or governing equations, nor do you need to have explicit assumptions that describe the underlying engineering processes.

In order to estimate all of the potential answers to issues in engineering or science, GA mimics the reasoning behind natural evolutionary processes. In contrast to GA, the majority of traditional methods follow a predetermined set of steps in order to get closer and closer to the best possible answer. The majority of the time, these algorithms start with a random guess at a solution. After that, they obtain a search direction depending on a specified transition rule. After that, a search in only one direction is conducted so that the best possible answer can be found. This kind of traditional optimization approach is ineffective in the desalination of water and the treatment of wastewater because the optimal solution is dependent on the starting solution that is chosen. In addition, it provides maximum and minimum values at the local level, whereas designers typically look for maximum and minimum values at the global level. While GA is capable of producing a Pareto set that identifies the point at which all feasible solutions are optimal and can anticipate them. In addition, a concise overview of artificial intelligence (AI) resolutions and strategies to overwhelm the disadvantages of traditional systems in many roles of PV systems is presented in Table 1.

**Table 1:** Overview of AI solutions and strategies for overcoming existing system drawbacks.

Traditional Algorithm	Practical use	Weakness of Traditional Algorithms	Solutions With Artificial Intelligence	AI Methodologies
Methods that are both predictive and stochastic	Monitoring, Maintenance	Outlier-sensitive	Substitute outliers with more appropriate values by utilizing Quantile Methods.	ML and DL
Kernel methods	Control/Maintenance	The results are probabilistic, and the training takes a lengthy period.	Predictability employs statistics to examine the frequency of probabilities and minimizes training time by solving sets locally	Regression Algorithms, Neural Networks and ML
Data Minimization Technique	Maintenance	Can only be utilized when clustering is present.	Filtering and Normalization replace data minimization	Memory- and model- Filtering ML
Randomized Probabilistic technique	Maintenance	complex computations	Symbolic reasoning is used to tackle difficult computational problems.	Logical -Neural Networks and Decision trees
Population based methods	Design control/maintenance	Slow convergence, complicated implementation	Pre-training with low learning rates for quick convergence	Machine Learning, Heuristic search

## 3 Utilization of artificial intelligence in PV- systems

**Fig. 2:**
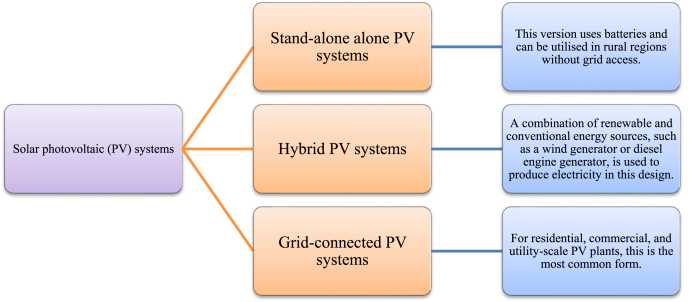
Different categories of PV systems [49].

PV systems are designed to convert the irradiance of the sun into electrical power. The big price of constructing PV systems is one of the primary drawbacks of these types of systems. Every research in this subject has attempted to enhance such systems while lowering their costs. The utilization of artificial intelligence algorithms has been demonstrated to play a pivotal function in augmenting the efficacy of solar panels. This study presents complete analysis on the application of artificial intelligence in modelling, sizing, controller, liability diagnostics, and output approximation of solar systems. It analyses the differences and similarities among AI and traditional algorithms for controlling individual type of application. Solar photovoltaic (PV) systems are composed of PV, batteries, converters, and inverters. On a worldwide scale, they are divided into the following three categories of system (see Fig. 2).

**Fig. 3:**
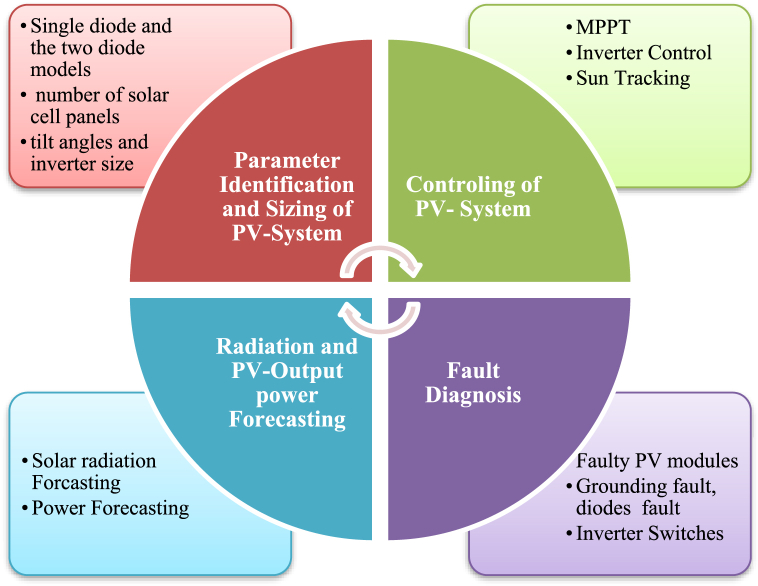
Parameters in PV Systems where AI-ML is applied.

In multiple ways of the sizing, modelling, and regulating of PV systems, AI algorithms are applied. The uses of AI in photovoltaic research broken into 5 primary categories see (Fig. 3). The next part will elaborate on each.

### 3.1 Selection of parameters for the model of solar cells

**Fig. 4:**
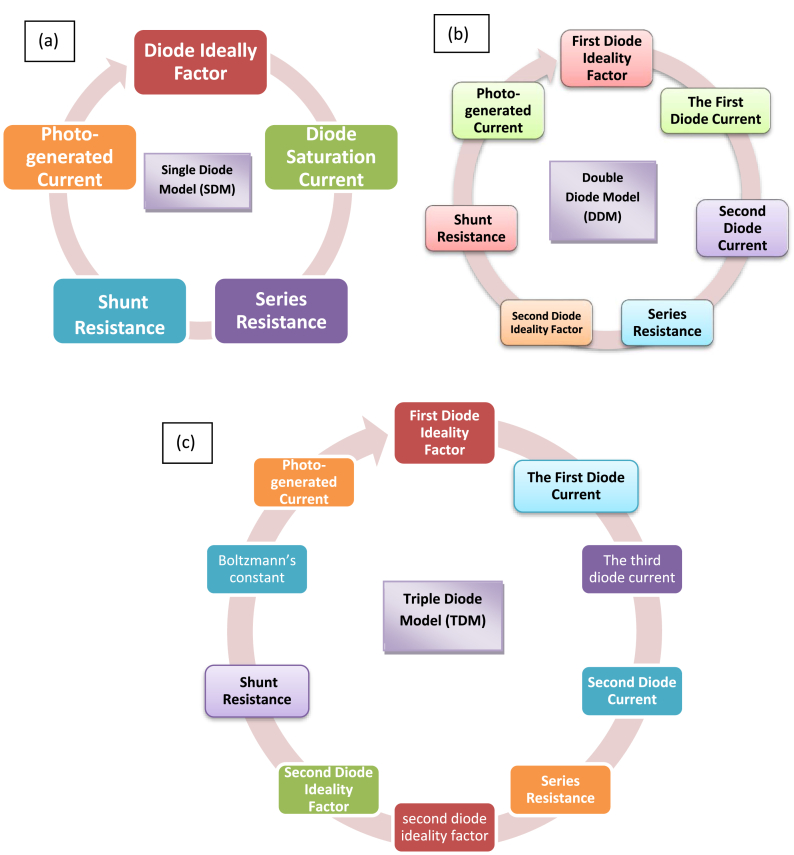
Parameters of each diode models, Redrawn: (a) SDM [52], (b) DDM [52], (c) TDM [52].

Within the realm of PV research, an exact modelling of PV cell is an essential component. In order to model a PV system, one needs to first theoretically model the system and before draw out the system's elements. The solar cell can be modeled using a Single-Diode Model (SDM), Double-Diode Model (DDM) or Triple-Diode Model (TDM) [50]. The parameters of each diode models are represented in Fig. 4. Since, SDM can be easily implemented with only five parameters; it is utilized extensively for the modelling of solar cells. Nevertheless, SDM is mostly imprecise in its description of cell function at low illumination conditions. Whereas, in DDM, which consist of an additional diode connected in series through the current. This additional diode has the potential to attain more precision than a model with a single diode, but because there are seven parameters, additional computations are required. The inclusion of other diodes together with the existing two diodes results in the creation of the TDM. When compared to the DDM, the TDM has an additional third parameters [51].

For the purpose of parameter identification of solar cells, there are a lot of traditional approaches described in the relevant literature. An analytical-numerical method is suggested for determining the 5 parameters of the SDM by Hejri et al. [53]. A first guess for the numerical solution can be derived from the analytic portion of problem. A pattern search approach is given by AlHajri et al. [54]. This method is applicable to SDM, DDM, and photovoltaic modules. However, orthodox approaches are incompetent of determining the characteristics of PV modules with a level of precision that is acceptable. Numerical methods, like the Newton-Raphson method [55], heavily rely on the first conditions that are selected. These methods also have a singularity problem and require a laborious calculation of the Jacobin matrix. In a similar manner, the non-linear least square (NLS) method presented in Ref. [56] is extremely sensitive to the initial values of the parameters and the bound constraints that are chosen. If the initial parameters aren't chosen correctly, the NLS optimization process could become stuck on a local minimum while it's trying to find the global minimum. Because of this, a large number of researchers began looking at AI methods for parameter detection.

In Karatepe et al. [57] describe a NN modelling approach for a SDM. On the other hand, in Askarzadeh et al. [58] propose an element identification using a Harmony-Search (HS) algorithm. According to the findings presented in the research, the effectiveness of the harmony search algorithm is significantly higher than that of simulated annealing and GA. El-Naggar et al. [59], the simulated annealing approach is utilized to search and find tool. In Askarzadeh et al. [60], an innovative method for resolving the problem of constraint identification based on a BMO is initiated. The findings demonstrate that the method has a performance that is superior to that of the PSO. In addition, parameter identification based on ANFIS rather than neural networks is described by Salem et al. [61]. The findings demonstrate that NN performs more effectively than ANFIS does. AIS is provided as a potential answer in Ref. [62], where it is contrasted with PSO and discussed. The findings of the study demonstrate that AIS performs more effectively than PSO and GA together. Table 2 provides a summary of some of the most modern algorithms that have been utilized in latest years for the purpose of PV parameter estimation problems.

**Table 2:** Modern algorithms utilized in recent years for the purpose of PV parameter estimation problems.

Reference	Algorithm	Diode models	Remark
[63]	Cuckoo Search Optimizer (CSO), improved CSO (ICSO) and modified CSO (MCSO)	SDM and DDM	ICSO achieves better accuracy and dependability than CSO and MCSO.
[50]	Supply-Demand-Based Optimization Algorithm (SDOA)	TDM	In the process of PV parameter extraction, SDOA is frequently used as a competitive optimizer.
[64]	Turbulent Flow of Water-Based Optimization (TFWO)	SDM, DDM and TDM	The suggested TFWO achieves close (IV) curves compared to other optimization techniques.
[65]	Harris Hawk Optimization (HHO)	TDM	The outcome presents that the recommended approach can quickly notice the electrical constraints of any marketable PV panel.
[66]	Gorilla Troops Optimization (GTO)	SDM and DDM	GTO is proven using a variety of irradiations and temperatures, all of which result in an extremely high degree of similarity among the emulated and investigational (IV) curves.
[67]	Forensic-Based Investigation Algorithm (FBIA),	SDM, DDM and TDM	The FBIA results are remarkably consistent because the SD of fitness values across 30 runs is fewer than 1 × 10^6^ for all three models.
[68]	Closed-loop PSO (CLPSO) and elephant herd-optimization (EHO)	DDM and TDM	The EHO is superior to the CLPSO with regards to the quality of the solutions it generates and the merging rates it achieves when viewed from the perspective of soft computing standards.
[69]	Metaphor-Less Rao-ii and Rao-iii Algorithms	SDM, DDM and TDM	As per the findings of the statistical analysis, the suggested algorithms, R-ii and R-iii, demonstrate a superior level of performance to those of well-established approaches.
[70]	Grasshopper Optimization Algorithm (GOA)	TDM	The usefulness of the GOA photovoltaic (PV) model is estimated by contrasting the results of the simulation with the outcomes of PV models that are based on other optimization approaches. The results are within a range that is considered acceptable. The suggested GOA can be used to optimize RE systems, and smart grids.
[71]	Improved Bonobo Optimizer (IBO)	SDM, DDM and TDM	All of the suggested IBO's results outperformed those of other algorithms when compared.
[72]	Grey Wolf Optimization (GWO)	SDM	GWO outperforms PSO in fitness. The model has the lowest I–V and P–V errors.
[73]	Slime Mould Algorithm (SMA)	SDM, DDM and TDM	Given the observations and comments, the suggested SMA may provide superior parameter estimates and merging speed, as evidenced in the converging curve for every PV model.
[74]	Adaptive Compass Search (ACS)	DDM	The ACS method can significantly increase the capacity to conduct global exploration by generating an adaptable sequence of exploration directions based on prior searching results.
[75]	Fuzzy Adaptive Differential Evolution Algorithm (FADE)	SDM	According to the findings, the FADE algorithm is an efficient way for evaluating the elements of PV module models and has a higher level of robustness when it comes to identifying parameters.
[76]	Genetic Algorithm Based on Non-Uniform Mutation (GAMNU)	SDM, DDM	The statistical outcomes states that the suggested method overtakes existing advanced algorithms in accuracy and reliability. The suggested approach can extract solar PV model parameters.
[77]	Modified-Stochastic-Fractal-Search Algorithm (MSFS)	SDM, DDM	RMSE values among models and actual data are 10^−2^ or 10^−3^. Therefore, suggested approach is utilized to estimate solar cell and PV module parameters due to its efficacy and practicability.
[78]	Northern Goshawk Optimization (NGO)	TDM	The outcomes of the simulation demonstrate that the NGO is higher to other competed optimization algorithms in terms of how quickly and accurately they converge on a solution.
[79]	Performance-Guided JAYA (PGJAYA)	SDM, DDM	The PGJAYA approach for PV module model parameters appears promising. Additionally, the PGJAYA method can be considered an effective strategy for dealing with several other optimization issues in the energy system.
[80]	Enhanced Gradient Based Optimizer (EGBO)	SDM, DDM	The findings point to the newly presented EGBO as being superior to the original GBO algorithm, and it does rather well when compared against some of the other approaches that are described in the relevant academic literature.
[81]	Coyote Optimization Algorithm (COA)	SDM, DDM	Both models had fitness standard deviations (STDs) less than 1 × 10^−5^. This shows the algorithm's consistent outcomes.
[82]	Marine Predators Algorithm (MPA)	SDM, DDM and TDM	The MPA achieves outcomes that are comparable to those achieved by other optimization methods described in the research literature. The suggested MPA has strong statistical support and convergence for a variety of operational situations, including those with low and high irradiance.
[83]	Supply-Demand-Based Optimization (SDO)	SDM, DDM and TDM	The SD of the fitness values are lower than 1 × 10^−18^, 10^−17^, and 10^−6^, respectively, for three models, which indicates that the SDO is superior. These values were calculated using a total of 30 runs.

### 3.2 Sizing of PV-panels

**Fig. 5:**
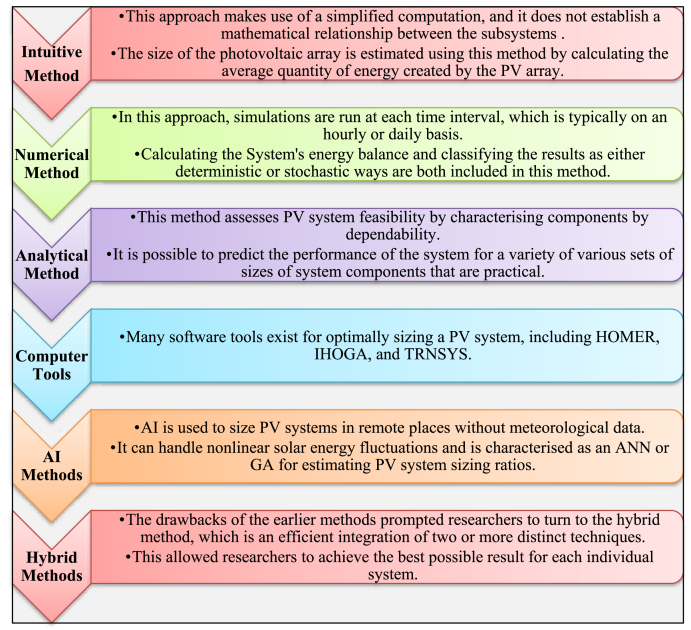
Different techniques to determining the size of a PV array.

Accurately sizing a solar photovoltaic ensures electricity quality and stability and maximises cost benefit during the system's lifetime [84]. Deciding the PV capacity, the size of PV module, the dimensions of the inverter, and the size of the battery are all elements of the PV sizing process [85]. The challenge of determining the exact count of solar panels, the optimal storing capability of the battery, exact positioning and slant positions of PV panels, and the optimal size of the inverter can be resolved by employing methods from artificial intelligence. Fig. 5 presents the several approaches that are available for determining the size of a PV array as well as the dimensions of battery in PV system [85,86].

An analytical approach was provided by Zanesco et al. [87] for the exact sizing of photovoltaic (PV) systems. This strategy was put to the test in 144 different cities across Brazil. Furthermore, In order to determine the exact size of freestanding photovoltaic systems in Malaysia, an innovative analytical method has been presented by Jakhrani et al. [88] Within the scope of this study, expressions have been formulated for the optimum size of PV arrays and useful battery storage capacities. Artificial intelligence approaches are now being used in research for the optimal sizing of photovoltaic systems since these techniques provide a higher level of accuracy than traditional methods. For the purpose of determining the optimal dimensions of a freestanding PV system, Khatib and Elmenreich [89] utilized a GANN. The authors of this study made use of an analytical technique in order to determine the sizing proportions for photovoltaic range along with the battery for the 5 locations in Malaya that were investigated. For this aim, data collected on an hourly basis from the climatological and load demand systems are employed. In the study referred by Mellit [90] genetic algorithm techniques and artificial neural network models are combined in a symbiotic fashion in order to accomplish the goal of optimising size coefficients for freestanding PV systems. The GA model was utilized to optimize the coefficients to decrease the price of the system. Subsequently, an ANN was trained with these inputs to discover the ideal coefficients for use in isolated places. In the same vein, an ANN is used by Kolawole [91] for the purpose of forecasting the ideal sizing factors for freestanding PV systems. The RMSE for the PV array size coefficient produced by the ANN was 0.046, while the RMSE for the battery storage was 0.085. The tabu search (TS) algorithm was used by Yoza et al. [92] to optimize the battery arrangement in house. During the two-part execution of the optimization challenge, the appliance scheduling part was optimized for operating expense, and the planning and operation part was streamlined for overall system cost. In a freestanding PV located in the Sfax region, Salah et al. [93] utilized FL to maximise both the surface area of the photovoltaic panels and the capacity of the batteries. MATLAB–Simulink is used to build fuzzy logic, and Inputs include load energy requirements and a monthly mean daily solar radiation. The technique produces PV panel contact area and battery capacity. Particle swarm optimization is implemented by Kornelakis and Marinakis [94] for the purpose of optimally sizing grid-connected PV systems. The data includes both the technical and economic aspects of system devices that are now available on the market, as well as meteorological data for the locations that were being considered. GA-based strategy was utilized by Zhao et al. [95] in order to optimize the unit sizing procedure for a freestanding micro-grids system located in China. In order to generate the sizing outcomes, an innovation operation approach has been adopted. This technique is centred on the coordination of energy storage. The technique that has been described was created in aim to decrease the release of LCC and pollutant emissions while simultaneously maximizing the production of energy. The sizing is determined with the help of daily data and straightforward models for the PV array and the batteries. When simple models are utilized, it is possible to have outcomes that are either over or under sized. In addition, the ANFIS model is created in in order to optimize the size coefficients of freestanding PV systems [96]. Based on the climatological data, the newly generated database contains sizing coefficients corresponding to two hundred different locations in Algeria. In addition, the ideal sizing criteria for these computed locations were produced, and they were based on the expenses of a solar panel. In comparison to the factors of the site's known size, the findings that the proposed ANFIS model provided were found to be the most accurate of those obtained from the many possible network topologies. The number of applications for AI algorithms in sizing is presented in Table 3. It is clear that NN and hybrid NN are the artificial intelligence algorithms that see the most widespread application for PV sizing.

**Table 3:** Number of applications for AI algorithms in sizing.

Reference	Algorithm	Parameters	Accuracy (%)
[96]	ANFIS	Sizing coefficients	98.5
[90]	GA & NN	Sizing coefficients	98
[89]	PSO	PV panel number, positioning, and tilt angle	–
[94]	Regression NN	Sizing-Curves	98.8
[91]	ANFIS-GA	battery size and solar cell count	92.5
[97]	bat algorithm	PV array, inverter module dimensions	96.4
[98]	Grey Wolf Optimization	optimize the size	–
[99]	PSO & GA	optimum grid connected PV size	–
[100]	PSO	range of the PV panel	–
[101]	Improved Harmony Search (HIS)	optimum size of PV/battery	

### 3.3 Controlling of PV-systems

In recent times, there has been a great deal of focus placed on the control of PV systems. In the published research over the course of the previous few years, numerous control objectives and controllers have been described. There is also the application of AI for the control of PV. Increasing the effectiveness of PV is accomplished over the application of various intelligent control approaches [102,103]. In this section, we will talk about the primary control components that are found in PV systems. There are three areas in which intelligent control approaches are utilized: MPPT [104], inverter controller [105], and sun tracking.

#### 3.3.1 Maximum power point tracking (MPPT)

**Fig. 6:**
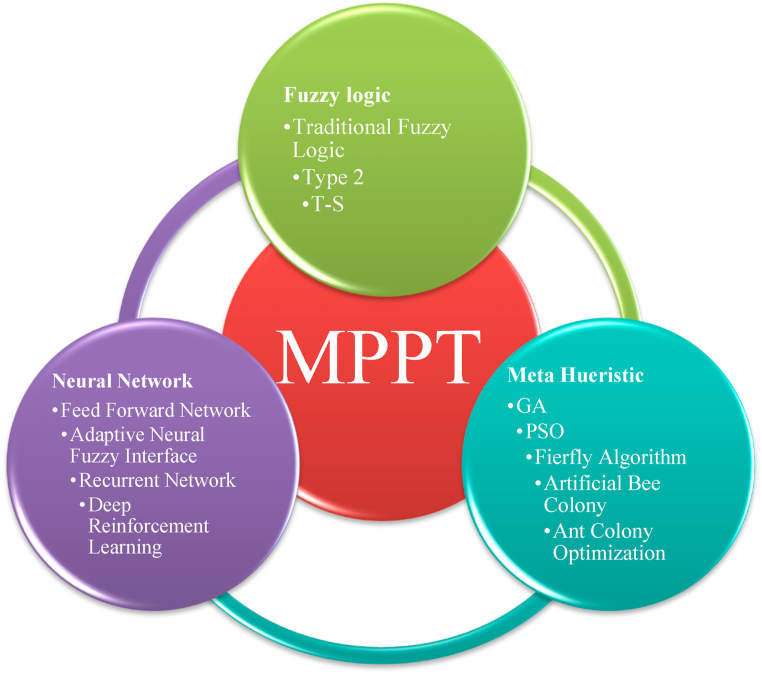
Different algorithms used In MPPT (Redrawn from [134]).

MPPT approaches are utilized in order to maximise the amount of power that may be strained from a PV and to expand the effectiveness of the installation [106]. The traditional MPPT approaches that are P&O [107], incremental conductance [108], fractional open circuit voltage [109], and current [110] are some of the most popular choices [111,112]. The ease of use and rapid convergence offered by these algorithms have contributed to their widespread adoption [113]. In recent times, systems for MPPT that are based on intelligent techniques such as PSO [114], genetic algorithms (GA) [115], fuzzy control [116], simulated annealing algorithm [117], neural networks [118], and firefly algorithm [119] have been developed. Fig. 6 demonstrates the variability of tactics that have been taken in the works in order to MPPT. The performance of AI algorithms has been shown to be superior to that of conventional methods. PSO for MPPT is presented in Refs. [114,120]. This research presents a controller for numerous photovoltaic arrays [121]. makes the suggestion that a neural network could be utilized to resolve the MPPT problem. MPPT is performed using an innovative ACO approach, which is found in Ref. [122]. Fuzzy logic's ease of use and lightning-fast reaction have contributed to the field of MPPT's meteoric rise in popularity. In Ref. [123], the author describes a FLC that can track the maximum power point under a variety of temperature and irradiance conditions. While in Ref. [124], an incremental conductance controller and a GA-optimized NN controller are linked to one another. An ANFIS-GA maximum power point tracking is recommended in Ref. [125]. The findings indicate that there is a relatively small amount of error in the MPP and the Optimal Voltage (Vmpp), as well as a higher competency of the recommended method in tracking MPP. Within the scope of study [126], a novel P&O variable step size that is based on a GA has been developed with the intention of enhancing the MPPT in PV systems. In study [1], fuzzy logic was used in an innovative way with artificial neural networks. This was accomplished by integrating genetic, particle swarm optimization and imperialist competitive algorithms in order to develop a solution for MPPT that was both efficient and effective. MATLAB software was used to carry out the approaches and evaluate their effectiveness. Kishore et al. [127] used TLABC methodologies to implement the dual TLABC methodology in MATLAB/SIMULINK. Additional statistics and stability assessment has been done on all approaches to improve accuracy. A novel dual TLABC algorithm is developed to eliminate GMPP deviations in this work. Four setups are used to evaluate the PV system. In the work by Kishore et al. [128] utilized GWO-DE, to record the global maximum peak power. Moreover, the system under consideration is constructed using the MATLAB/Simulink program [129]. The system is tested in different atmospheric conditions and compared to others. The hybrid GWO-DE algorithm surpasses previous methods in convergence time, precision extracted power, and efficiency. In the similar vein of the research Aljafari et al. [130] have suggested a novel hybrid MPPT that utilizes an BO algorithm to effectively solve the issue of fluctuation and its transformations. The study has offered a performance comparison and analysis of the butterfly optimization algorithm, GWO, and PSO based MPPT methodologies. The investigational consequences demonstrate that the suggested strategy outperforms standard approaches in terms of adaptation, effectively reducing the convergence of load variation and minimizing the occurrence of frequent exploration and exploitation patterns. PV systems are susceptible to several environmental disruptions, one of which is partial shade, a disturbance that negatively impacts the photovoltaic system's properties. Hence, it is imperative to establish a comprehensive analytical framework for PV systems in order to examine the optimal MPPT technique. A rigorous analytical model of the PV system under partial shading circumstances (PSC) is constructed by Ref. [131], considering the impact of both series and shunt resistance, in accordance with its necessity. The findings demonstrate that the suggested model has a immense quantity of reliability, rendering it suitable for accurately representing a wide spectrum of solar systems, encompassing both independent and grid-integrated configurations. Nevertheless, the efficacy of the suggested mismatch loss reduction technique has the potential to augment the power conversion rate [132]. B. Aljafari et al. [133] proposes a PV array configuration in a calcudoku puzzle arrangement in order to mitigate the effects of the partial shadow phenomenon. Though the calcudoku puzzle pattern bears resemblance to the sudoku puzzle pattern, the PV panel locations in the proposed work are determined through the application of mathematical relations. Additionally, there is no repetition of the same number in the corresponding column and row. The efficacy of the proposed system is assessed in a 9 × 9 photovoltaic arrays while considering all eight potential shading patterns. As part of the investigation into the methodologies' efficacy, a comparison was made between them regarding the stability, speed, and complexity of their respective implementations. Table 4 provides a concise breakdown of the many algorithms that are available.

**Table 4:** Concise breakdown of different algorithms for MPPT.

Method	Reference	Remark	Validation method
**Artificial Neural Network (ANN)**	[135]	In order to find the MPP quickly and accurately, a new MPPT technique established on artificial neural networks has been proposed.	Experimental parameters such as Voltage, Current, Radiation Intensity are taken to train ANN Model. The model is best suited in field-programmable gate array.
	[136]	When utilizing an ANN-based MPPT controller, the output voltage is more stable and has fewer oscillations than when using other controllers. The controller is more reliable overall and operates at a higher speed.	The performance is compared with perturb and Observe method and validation is done.
	[137]	In order to train neural networks, an algorithm called error back propagation is utilized. The NN has the benefits of quickly tracking the MPP.	ANN obtained results are validated by using standalone PV Panel connected to boost dc-dc converter.
	[138]	In this paper, design and analysis of a MPPT controller for a solar structure implemented in an ANN. The results reveal that ANN-based MPPT provides good performance under consistent and rapidly changing environmental circumstances.	PV cell is modeled with Cuk converter and output is analysed with ANN results.
	[139]	This research presents a Rprop–NN–based technique for PV-MPPT.	The existing studies cases are used for comparison of results obtained from Rprop-NN model.
	[140]	A low-complexity MPPT technique that is established on the neural network (NN) model of the solar module is presented in this research.	The calculated power output is compared with results obtained from proposed algorithm.
**Fuzzy Logic Controller (FLC)**	[141]	In this research, a P&O-based fuzzy logic MPPT controller that is tailored for rapidly changing insolation circumstances is presented. The power generated by MPPT controllers that make use of FLC is superior to that generated by conventional methods.	The power generated by MPPT controllers that make use of FLC is superior to that generated by conventional methods.
	[142]	In this article, the author presents the design and analysis of an isolated PV system that makes use of a push-pull converter and an MPPT algorithm that is built on fuzzy logic. The results of the simulation demonstrate that the proposed methodology is capable of tracking the MPPT in an effective manner.	The hardware PV Cell of 250W prototype is used to validate results getting from MPPT algorithm
	[143]	This article suggests employing a fuzzy logic controller (FLC) to run a recommended PV control system at the MPP of the array for each moment.	The results are compared with exiting literature.
	[144]	For a PV system that is linked to the grid, a unique MPPT method that is built on fuzzy logic (FL) has been presented. The proposed method works well with grid-connected PV systems, achieving 99.6 % efficiency.	The experimental results at different irradiation levels are used for validation purpose.
	[145]	This work provides a modified MPPT algorithm for multi-peak PV arrays that operate in partial shadowed situations. The fuzzy logic control is the basis for this proposal. By comparing the suggested method to particle swarm optimization and hardware experiments, its robustness, accuracy, and stability are confirmed.	The experimental results using one-diode PV model is used.
**Particle Swarm Optimization (PSO)**	[146]	The approach reduces steady-state oscillation (to almost zero) once the MPP is discovered. The proposed approach can track MPP in harsh environmental conditions. Due to the simplicity of the algorithm and the ease with which it may be computed, its implementation on a microcontroller with a lower cost can be accomplished.	The experimental result regarding voltage, current, duty cycle is obtained under similar conditions as that of simulations.
	[147]	This research uses an enhanced PSO algorithm to track PV MPP. To increase tracking speed, PSO particles are given an initial value defined by the I–U and P–U curves.	The improved PSO results are compared with conventional PSO in order to check feasibility of newly proposed algorithm
	[148]	In this research, a novel benchmark test is presented in order to assess the effectiveness of various EA-inspired MPPT algorithms in comparison to a variety of shaded curves.	The experimental PV curve is compared with curved obtained from PSO algorithm for validation.
	[149]	In this paper, an IPSO-based MPPT approach for tracking MPP was presented. The findings showed that the suggested method has a high convergence speed, and the structure of the enhanced MPPT algorithms is very simple.	The effectiveness of novel techniques is compared with previous published results related with incremental conductance.
	[150]	The aim of this work is to provide a velocity of PSO-based Levy flight (VPSO-LF) for global MPPT of PV systems operating with PSCs. Under a variety of PV array configurations, it has been discovered that the results obtained through the use of VPSO-LF are superior to those obtained via the use of standard PSO and hill-climbing algorithm.	The results are validated with experimental results obtained from PV Simulator integrated with boost converter.
	[151]	PSO is employed to identify the optimal sliding mode controller (SMC) gains for P&O algorithm.	The conventional fixed step P&O and Solarex MSX-60 module results are compared with proposed PSO algorithm.
**Ant colony optimization (ACO)**	[152]	An ACO technique is developed in this work. This technique effectively follows the global peak and enhances the performance of PV arrays as a result.	The conventional fixed step P&O results are compared with proposed ACO algorithm.
	[153]	For maximal power point tracking in this investigation, a brand-new bio-inspired technique known as ACO NPU was utilized.	The results are compared with existing literature related with PSO & ACO and conventional P&O results.
	[154]	The objective of this research is to provide a technique for controlling the speed of a SRM that is powered by a PV system. In order to find the ideal PI parameters, an approach known as ACO is being utilized.	The optimization problem is built for speed controller motor and result are validated with analytical results obtained from optimization problem.
	[155]	This study adapts ant colony optimization to MPPT in photovoltaic (PV) systems. The idea is represented appropriately, and MPPT curves in a few different PV systems are simulated.	PV Cell with four series-four parallel is used in experiment to validate results related with maximum power tracked, convergence time.
**Genetic algorithm (GA)**	[115]	This research uses GAs-based MPPT to increase PV system convergence, speed, and accuracy. The suggested technique tracks the global MPP effectively, which is important for partial shading.	The results are validated with test model built in facility of LIAS laboratory, Poitiers, France.
	[156]	This research provides a MPPT method that is based on a GA for a PV array that is coupled with a BSU The MPPT strategies based on GA have been compared to the conventional PO algorithm, and they have been found to be competitive with it.	Conventionally P&O is used for comparison of results obtained.
	[157]	GA optimized ANN- MPPT technique is suggested. The primary goal of this design is to do away with the dc–dc converter, and the losses that come along with it. The efficacy of the suggested strategy has been demonstrated through the use of both simulation and experimental findings.	The 60W PV system is used to construct experimental setup and results are used for comparison with algorithm obtained results.

MPPT approaches utilize controllers to maximise power output from PV setups. There are numerous MPPT approaches for running PV modules at maximum power. The effectiveness of this approach relies on its capability to track speedily varying meteorological situations. Techniques for tracking in PSCs are classed based on their nature. Discussion covers all classified methodologies, categorized as Classical MPPT, Intelligence MPPT, and Optimization MPPT [158].

Under homogeneous irradiation conditions, photovoltaic systems are highly efficient as they generate only one GMPP. However, it should be noted that these algorithms exhibit rapid oscillations around the MPP, which subsequently leads to power loss. Moreover, it is important to note that the aforementioned traditional methodologies fail to consider the impact of PSC, resulting in an inability to accurately monitor the MPP. Comparison of classical algorithm-based MPPT approaches represented in Table 5.

**Table 5:** Comparison of various MPPT Control algorithm.

Reference	Technique	Tracking speed	Accuracy of tracking	Sensing parameter	Complexity	Efficiency %
[161]	Constant voltage	Slow	Slow	Voltage	Simple	73
[162]	Adaptive reference voltage (ARV)	Medium	Medium	Current & voltage	Simple	98
[163]	fractional open-circuit voltage (FOCV)	Slow	Low	Voltage	Simple	93
[163]	perturb and observe (P & O)	Slow	Medium	Current & voltage	Complex	98
[164]	Ripple correlation control (RCC)	Fast	High	Current & voltage	Complex	96
[160]	ANN	Medium	High	Irradiance & temperature	Simple	98
[165]	FLC	Fast	High	Current & voltage	Medium	97

Intelligence-based strategies, as discussed in previous studies [158,159], encompass many methodologies such as FLC, ANN, SMC, MPPT algorithms based on the Fibonacci series, and MPPT algorithms based on the Gauss-Newton approach. These strategies are designed to address dynamic weather situations with a high level of precision. The tracking efficiency and speeds exhibited by the system are remarkably great. These methods are also characterized by significant control circuit complexity and extensive data processing required for system training prior to implementation. The FLC methodology is a notable method that does not necessitate extensive understanding of the system for the execution of MPPT. The use of SMC, an advanced technology, is facilitated by its ability to track speeds at higher rates.

PSO, GWO, CS-based cuckoo search, ACO, and ABC are optimization-based methods [160]. These methods also seek true MPP in dynamic environments. PSO is a quicker tracking algorithm with less oscillation. These strategies are easy to apply using low-cost microcontrollers. Like a wolf hunting prey, GWO can find the best working location faster. This GWO is the finest system-independent evolutionary method. Bio-inspired CS-based MPPT employs brood parasitism and levy flying approach to find the optimal MPPT. Emerging methods like ACO and ABC use evolutionary-based algorithms. They use fewer temperature and voltage sensors than traditional methods.

#### 3.3.2 Inverter control

In photovoltaic (PV) systems, there is a stage that comes after the DC/DC converter and is called the DC/AC inverter. It is up to the inverter to generate the three-phase alternating current power that the load requires. The regulation of the inverter's AC output power and frequency while maintaining a minimal level of harmonic distortion is the goal of inverter control. The implementation of an inverter control algorithm allows for control of the switches that are contained within the inverter. AI can increase the accuracy and reaction time of the inverter controller to transitory defects compared to conventional controllers, which use PI and PR algorithms [105]. An implementation of a FLC for a single step h-bridge multiple level inverter is found in Ref. [166]. S-function deals with PSO optimized FLC for regulating PV systems is provided in Ref. [167]. An ANN-controlled inverter is constructed and replicated in Ref. [168]. In order to adjust the PI controller settings in the inverter system, the PSO technique was applied in the interests of reducing the amount of error produced by the voltage regulator and current controller schemes [169]. An off-grid PV inverter is used in a research project [170] to generate three-phase power to feed the local load. This inverter is regulated by an optimized FLC that makes use of PSO to regulate the output of the PV system. The suggested controller has been tested out on a variety of loads, including nonlinear loads, inductive and resistive loads, and pure resistive loads. The results of these tests have been compiled and analysed. In the work that has been proposed in Ref. [171], a neural network is utilized in order to modify the inverter gain settings. The inverter is built with a conventional PI controller, and its gains are trained with grid variables such as voltage and current. The output of the PI controller is also calibrated with regard to the grid. The neural network makes predictions about the gain elements of the PI controller depend on the variations in the grid side parameters, and the PQ of the grid side has been improved. In the [172], RBF neural network is utilized to regulate 3-phase grid linked inverter. In a research [173], ANFIS- MPPT controller for solar systems that utilize fewer sensors is presented. The error signal was provided by the ANFIS controller, and a fuzzy controller had to be tuned in order to create the correct duty cycle for the 2-switch fly back converter. The findings demonstrate that the MPPT controller that was developed for a two-switch fly back inverter is capable of monitoring the supreme power output regardless of the surrounding atmospheric conditions.

A novel control method for multilayer DC link inverters (MLDCLI) effectively addresses the issue of reduced solar radiation due to partial shade of individual photovoltaic (PV) modules. The algorithm proposed by Ramachandran et al. [174] utilizes a hybrid MPPT and pulse width modulation technique to regulate an MLDCLI system in the face of fluctuating solar energy. The MLDCLI is assessed using phase opposite disposition (POD) multiple of carrier pulse width modulation (MCPWM). This approach optimizes the power extraction from individual shaded PV sources without impacting the performance of other PV sources. Microcontroller-based prototypes, which have been evaluated using MATLAB/SIMULINK system generators, demonstrate significant enhancements in voltage quality for standard output levels. The filter is used to receive the output of the inverter in grid-connected applications.

#### 3.3.3 Sun tracking control

A sun tracking controller is required for the PV system. This controller guides the PV panel in the direction of the sun. It has been demonstrated through a significant amount of study that tracking the sun improves the efficiency of photovoltaic systems. Single-axis sun tracking and double-axis sun tracking are the two varieties of controllers that are available for sun monitoring. There is a presentation of a genetic algorithm optimized dual-axis sun tracking system in Ref. [175]. In Ref. [176] the authors suggest an intelligent sun tracking system that may be used with dual-axis sun tracking. Two different control algorithms are reported in Ref. [177] in order to follow the sun. The Firefly algorithm (FA) is built into the Arduino Mega microcontroller by Ref. [178], and its purpose is to control the tracking of the sun's location by the solar panel. This is done in order to ensure that the solar panel is able to engross as much solar energy as conceivable, thereby producing the maximum amount of electrical energy. It is possible to draw the conclusion that the Firefly algorithm offers a workable solution to the optimization issues that arise with solar trackers. The primary purpose of the study carried out by Ref. [179] is to determine which optimization strategy will provide the most beneficial outcomes for the position control of the Sun Tracking System. The PID controller of this system is tuned using a variety of techniques, including GA, PSO, and TLBO, to control the position of the system. These procedures were ultimately carried out to select the effective technique for PID tuning, which is used to regulate the position of the sun tracking system. The TLBO-tuned PID controller performs the best and most reliably, according to the findings [180]. suggested an intelligent sun tracking system that combines a dual-axis sun tracking system with a MATLAB-created MPPT to switch between dual-axis, one-axis, and stationary solar panels. Using the swarm intelligence approaches of PSO, FFA, and CSA, the study work carried out by Ref. [181] considered the design of an ideal PID controller for a dual axis sun tracker system. This was done to attain the best possible outcomes. In order to tune the PID controller for both axes, researchers have used the three-swarm intelligence-based met heuristic approaches. It has been determined, on the basis of the data and observations, that CSA is more successful than PSO.

### 3.4 Radiation and PV-output power forecasting

In recent years, grid-connected photovoltaic (PV) installations have increased. Because of this, it has become an increasingly important issue to have correct forecasts for the quantity of power that is supplied to grid. The amount of power that PV systems generate is depends on the irradiation of the sun and the cloud cover that is present at the time [182].

#### 3.4.1 Forecasting the amount of solar radiation

Irradiance from the sun is the primary source of fuel for photovoltaic (PV) systems; hence, studies that forecast solar irradiance are extremely helpful to the operation of power grids and systems that contain PV. Forecasting solar irradiance by employing ANN, SVM, k-NN, and DL are described in Ref. [183]. In the most recent decades, advancements in technology have led to the rise of AI, which has quickly become quite popular in virtually all engineering sectors [184]. According to the findings of the earlier studies, it has been demonstrated that the algorithms used by AI are more accurate than those used by empirical models [185,186]. For instance, Quej et al. [187] forecasted day-to-day solar radiation data for six stations in Mexico by utilizing three different ML approaches. These algorithms are: Data on alien solar radiation, rainfall, and lowest temperature, were utilized by the authors throughout the training process for the algorithms. In this study the best results were obtained using SVM, which had RMSE values of 2.578, MAE values of 1.97, and R^2^ values of 0.689. In a different research study, Marzo et al. [188] attempted to forecast the daily worldwide solar radiation that was measured at 13 distinct locations. In that particular piece of research, the authors exclusively employed ANN as a form of machine learning algorithm. The ANN algorithm that was utilized to train for the study was trained with the alien solar radiation. The best outcomes, according to the calculations, were rRMSE equal to 13 %, rMBE less than 4 %, and r equal to 0.800 (R^2^ equal to 0.64). Mehdizadeh et al. [189] looked at the daily sun radiation and employed three distinct models namely, Gene Expression Programming (GEP), ANN, and ANFIS. The ANN model produced the top outcomes. For the purpose of solar radiation forecasting, Messai et al. [190] employs a novel multiple parameters neural network. In order to provide accurate predictions of solar irradiation, fuzzy logic and neural networks are utilized. Chen et al. [191], the output of the suggested method follows the real values even though the environment conditions are constantly changing. For the purpose of hourly irradiance forecasting, support vector machine modelling is suggested to be used in Ref. [192]. Discussions in Refs. [193,194] offer a comprehensive summary of the many DL models that can be used for estimating solar irradiance. The wavelet decomposition-based networks are utilized in the process of constructing the approach for solar irradiance forecasting that is described by Wang et al. [195]. In Ref. [196], the ANN model is established by tailoring it to a specific period of the year in order to provide correct method of predicting. This is done in order to make the model more predictive. The created method is supplemented by the Pearson correlation method in order to supply the ANN model with the best appropriate set of inputs. This enhances the model's computing power, allowing it to produce more accurate predictions even in the face of significant anomalies and dynamically shifting conditions. Ciabattoni et al. [197], a RBF is utilized to make predictions regarding the irradiation of the sun. For the purpose of estimating solar irradiance, the suggested algorithm is contrasted with ANN as well as other tried and true approaches [198]. presents a complete evaluation on the application of ANN in the process of estimating solar irradiation. The usage of ML classifiers, such as the multilayer perceptron NN [199], the Naive Bayes technique [200], and the k-nearest neighbor NN [201], evolutionary algorithms [199], frequently used for solar radiation predicting in addition to the DL and NN approaches.

#### 3.4.2 Forecasting PV-output power

Solar photovoltaic (PV) power generation is extremely susceptible to variations in the surrounding climate [202]. Then, techniques of prediction are essential to reduce the imbalance among the expected power and the actual power generation, which is necessary to support the operation of the power system [203]. There are two distinct types of forecasting models, which are known as indirect models and direct models. In models for indirect weather forecasting, a weather prediction serves as an input for photovoltaic (PV) simulation software, which then generates an energy forecast. In the meantime, direct models make their predictions based on historical information of the weather and the amount of power produced by PV [202]. In Ref. [204], a method for estimating the output power that makes use of both AI and WT techniques is proposed. The WT is used in the suggested method to have a significant effect on PV power time-series data, and AI techniques are utilized to catch PV variation in a more effective manner. In the process of forecasting solar output power, fuzzy logic controllers are also utilized by Yazdanbaksh et al. [205]. In this work, a new method of forecasting that is based on complex fuzzy logic is contrasted with two existing ML approaches for making predictions. When compared to other methods, the one that was suggested provided more precise projections of power output on a simulated solar cell 1 min in advance [205]. Furthermore, a dynamic neural network is utilized to make a prediction regarding the photovoltaic yield in Ref. [206]. This research work addresses two practical methods for estimating the amount of electricity that will be generated by PV plants that are connected to the grid. The first model is constructed on the basis of a cyclical ARIMA time-series examination and it is enhanced even further by the incorporation of short-term solar radiation predictions resulting from NWP models. Vrettos et al. [207], researchers introduced a parallel architecture hybrid SARIMA-ANN model for PV power forecasting. This model combines the best features of the SARIMA model and the ANN model. Based on the findings, the hybrid approach is 10 % more effective than using the different models individually for making forecasts. The performance of several ML models that anticipate the quantity of power created by PV is analysed by Visser et al. [208]. The models for forecasting are constructed by making use of historical data of PV power as well as predictions of the weather. In Ref. [209], a novel idea for solar generation forecasting is proposed. The proposal is based on exploring meteorological aspects from PV model. The procedure is carried out in three stages, which are the modelling of PV systems, the application of ML techniques to the process of mapping meteorological variables using solar power, and the correction of the forecast. Huang et al. [210] presents a PV output forecast that is based on weather prediction. In order to categories historical generation data, the K-means clustering algorithm is utilized, and the correlation analysis approach is utilized to minimise the dimension of the inputs. The problem of solving the prediction model can be handled by taking into account the long-short memory NN in conjunction with the attention mechanism. A back-propagating NN for power forecasting of PV systems is provided in reference number [211]. By integrating a forecast adjustment stage, one is able to obtain a more accurate prediction of the generation of PV. As a result, an additional term has been added in order to achieve better outcomes. This function can be found in Refs. [212,213]. Based on the research presented here, one may draw the conclusion that artificial intelligence-based models are the most often used predictive approaches for photovoltaic (PV) generation. ANN, SVM, ML, and regressive methods are some examples of techniques that fall under the umbrella of artificial intelligence. Additional classifications include statistical, physical, and hybrid models.

### 3.5 Fault diagnosis in PV

**Fig. 7:**
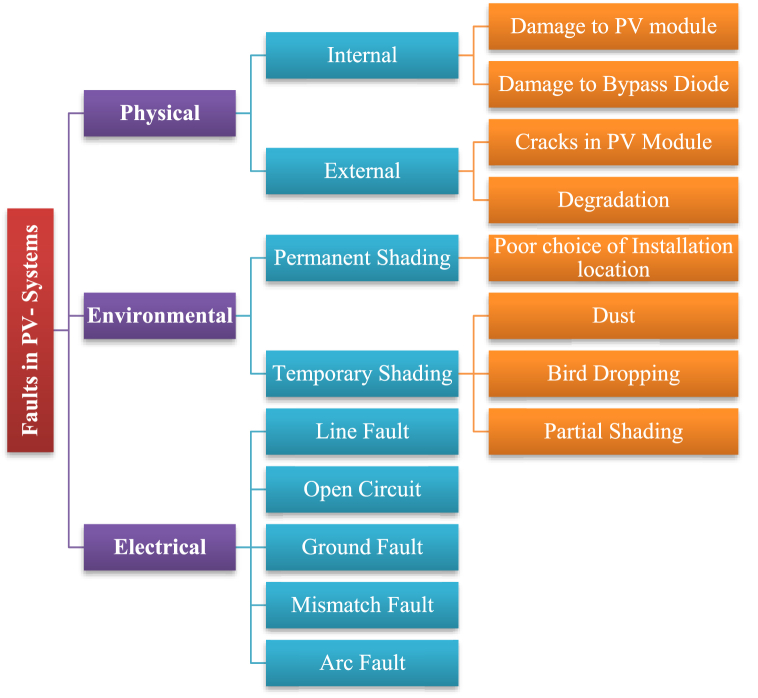
Various types of faults associated with PV systems [222] **(Redrawn and adapted with permission from ELSEV. B.V. with LIC. No 5517580437414)**.

The vast majority of photovoltaic systems are designed to function in the harsh climate of the outdoors. Working in such a situation increases the probability that the PV system will have malfunctions. These flaws could be caused by the ageing of the material, shadowing, a short circuit, or an open circuit. PV plants should be properly protected from the many types of failures to provide steady production, availability, dependability, and security [214]. There are several different standards that try to protect photovoltaic plants and reduce the risk of faults. On the other hand, certain flaws go undetected and can result in major complications, such as an increased danger of fire. A photovoltaic (PV) system may have a variety of problems, each of which may be classified according to one of several different criteria represented in Fig. 7. These kinds of flaws are separated into three categories by us: physical, environmental, and electrical [215]. Nevertheless, faults can also be categorized such as their location and their structure [216]. Damage, cracks, and deterioration are examples of some of the more common types of physical defects that can occur in PV modules. These faults can originate from either the inside or the outside of the module. The ageing effect, which is also a physical process, is another factor that contributes to the failure of PV systems. Among the environmental flaws are the accumulation of mud and dust, the droppings of birds, and the temporary shadowing. Open circuit, line-line, and ground faults are the three types of electrical faults that can occur in photovoltaic (PV) modules, arrays, or the entire system. Open circuit failures are created when wires in a single or several branches of a photovoltaic circuit get disconnected from one another. A comprehensive evaluation of photovoltaic defect detection and monitoring systems is contained in Ref. [217]. This study reviews monitoring approaches for major system breakdowns. In another work [218], the authors discuss PV system tracking, evaluation, and power prediction advances. The greater part of tracking and fault detection strategies make use of inverter level monitoring due to the ease with which data can be collected and processed. On the other hand, the majority of these methods are unable to locate faults within a PV string because they do not provide adequate visualization. There have been many different faults diagnostic techniques proposed, and some of these techniques are based on simulations for performance analysis [219] statistical analysis [220], and the use of current and voltage measurements, all of which are dependent on accurate modelling and processing in order to detect the fault. Various environmental factors, such as hotspots, partial shade, and minor flaws, might limit the effectiveness of a solar PV system. This results in the PV system experiencing irreversible failure and power reductions. In a study by Alwar et al. [221] enhances the power output of a solar system that is partially shaded by implementing a fault classifier that utilizes thermal image analysis together with a reconfiguration method. This practice is particularly valuable for photovoltaic (PV) systems used in health monitoring, as it effectively reduces the impact of partial shading and small malfunctions. The required task was effectively carried out using a 4 × 5 photovoltaic (PV) arrays.

**Fig. 8:**
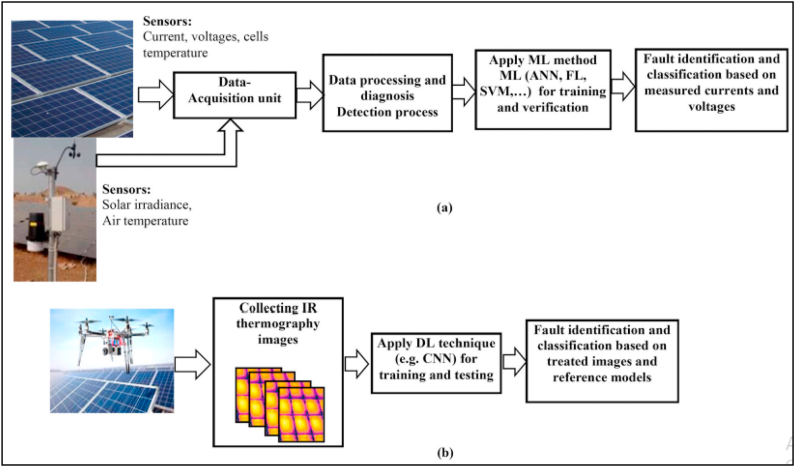
(a) I–V-based machine learning and [5] (B) Thermography-based deep learning frameworks for defect identification and classification [5] **(Adapted with permission from ELSEV. B.V. with LIC. No 5517450814386)**.

The application of ML and more recently DL, are both examples of methods that rely on AI and are used for fault recognition. DL is primarily utilized for fault diagnosis depends on infrared and electroluminescent pictures, whereas ML is primarily utilized for procedures that rely on I–V curves, currents, voltages, and other constraints. These methods require a large database to train and evaluate categorization and recognising models. The basic operation of these various methods is illustrated in Fig. 8. AI-based methods recognize, categorise, and characterise problems. These procedures can also fix defects with similar signs or indications [5].

In recent times, a variety of machine learning methods have been used to the FDD of PV systems. The decision tree based fault detection that is proposed can be found in Ref. [223]. The discovery and diagnosis of defects like these are extremely important roles that artificial intelligence performs. The method of training the model can be described as being simple and easy to put into action. The trained model demonstrates strong detection performance (up to 99.98 %) and classification accuracy (up to 99.8 %), both of which may have intriguing applications in the real world. A learning strategy using expert systems for diagnosing a PV system was developed by the authors [224]. In order to diagnose the shading effect, the technique that was devised is applied. The methodology was tested through simulation as well as experimentation. The procedure is straightforward, and it achieves the desired results; however, its viability can only be established in the context of the shading effect. In Ref. [225], a very simple coding procedure BPNN was presented. In general, the approach delivers decent correctness; however, the method was not evaluated with actual data. A household PV system is given an intelligent failure detection approach in Ref. [226], which is constructed using a modified artificial neural network (3.15 kWp). In order to calculate the power output, the Solar Pro simulation software was utilized. The simulation reveals that the suggested method is capable of precisely and rapidly identifying malfunctioning PV modules, also known as shaded PV modules. A proposal for on-line monitoring and defect diagnosis using BPNN that have been improved using a genetic algorithm can be found in Ref. [227] A comparison is made in Ref. [228] between the BPNN, the WNN, and the improved WNN. The comparison demonstrates that the upgraded WNN performs more effectively than its competitors. In the study referenced in Ref. [229] a two-layer NN is integrated with traditional numerical methods in order to determine which of 6 types of errors is present.

Ganesan et al. [230] introduced a novel method for detecting faults, which involves utilizing the fewest possible sensors to identify faults in 8 × 4 photovoltaic arrays arranged in bridge and honeycomb patterns. The suggested method utilizes voltage measurements between adjacent strings to detect open-circuit and line-to-line defects in a solar PV array. The magnitude measured by the sensor determines whether the problem is an open-circuit fault or a line-to-line fault. The current methods have been unsuccessful in identifying open-circuit and line faults in specific circumstances. However, the suggested approach is able to detect open-circuit and line faults in all situations, regardless of whether the configuration is bridge or honeycomb. The average power improvement before and after fault clearance in the conventional bridge configuration approach is 64.88 %, while in the suggested method, it is 65 % for open circuit. The average power in bridge configuration for the suggested approach increased from 65.88 % to 75.04 % in the case of a short circuit. The mean power amplification prior to and subsequent to fault clearance in the current approach in honeycomb configuration has an efficiency of 52.91 %. In the proposed technique, the open circuit efficiency is 64.66 %. Chine et al. [231], an implementation of a NN for fault diagnostics using field programmable gate arrays (FPGA) is presented. Wu et al. [232] classified short circuits, shadows, and ageing in a photovoltaic (PV) system using a RBF-ELM network. The findings demonstrated that the typical categorization rate is 93.55 %. In this particular study, only the findings of simulations were presented. For the purpose of fault classification in PV arrays, an approach that makes use of theoretical I–V curves and fuzzy classifiers was developed [233] The fault detection method that was created is dependent on the various voltage and power fluctuations that occur inside the system. Annually, every individual solar panel has a reduction in efficiency ranging from 0.5 % to 1 %. The deterioration of solar panels occurs as a result of both environmental and electrical deficiencies. An expedient and precise identification of environmental defects mitigates the harm inflicted by those defects on the panel. Deep learning, specifically convolutional neural networks, have achieved remarkable outcomes in several applications in recent years. The primary objective of the study performed by Selvaraj et al. [234] is to optimize the performance of pre-trained convolutional neural network models, with a specific emphasis on AlexNet, GoogleNet, and SqueezeNet. SqueezeNet is employed to train thermal pictures of solar panels and classify environmental defects, based on performance criteria. The acquired results demonstrate that SqueezeNet achieves a notable testing accuracy of 99.74 % and an F1 score of 0.9818. These outcomes indicate the model's effectiveness in detecting environmental problems in solar panels and assisting users in safeguarding the panels. The most common FDD algorithms for PV plants are shown in Table 6. The examined papers are compared in terms of their fault detection, identification, and localization capabilities as well as other metrics such as cost, faults explored, PV plant capacity, and algorithm type.

**Table 6:** Most common FDD algorithms for PV plants.

Reference	Type of Algorithm	Function	Type of Fault	PV-System used	Accuracy
[227]	BPNN-GA	Detection	local material ageing, shadowing, open/short circuit	PV Array	–
[235]	Fuzzy logic	Detection	Arc fault	PV Array	96 %
[236]	LAPART algorithm	Detection	Power Reduction	PV Module	86 %
[237]	fuzzy inference system	Detection & identification	DC side short-circuits	PV Array	94 %
[238]	neuro-fuzzy classifier	Detection & Classification	series losses, faulty by-pass diode, blocking diode	PV Array	90–98 %
[231]	ANN	Identification	By-pass diode short circuit, Connection fault	PV System	–
[232]	RBF-ELM	Classification	short circuit, ageing	PV Array	88.5 %
[239]	M-SVM	Identification & classification	Degradation, line-to-line fault	PV Module	98 %
[240]	PNN	Detection & Classification	short circuit, disconnected string	PV Array	100 %
[241]	fuzzy C-mean (FCM)	Detection & Classification	short, open, partial occlusion, and other defects	PV Array	96 %
[242]	decision tree	Detection & Classification	string, short-circuit, or line-line fault	PV Array	99 %
[243]	Improved GA	Identification & localization	short-circuit,	PV String	95 %
[244]	random forest (RF)	Detection & Classification	line faults, deterioration, open circuit, fractional shading	PV Array	99.13 %

Table 7 provides a summary and comparison of previous studies that have concentrated on the utilization of data-driven analytical models for the identification and categorization of photovoltaic (PV) module deteriorations and flaws. Additionally, the machine learning and deep learning models utilized in this study are described. The efficacy of ML and DL techniques in analysing PV module degradations is evident from the data shown in Table 7. Furthermore, according to the data presented in Tables 7 and it can be observed that the different kinds of neural networks (NN) have been widely embraced as they offer a high level of efficacy and accuracy, particularly in the domain of picture classification. The impact of data preparation on model performances is evident.

**Table 7:** Comparison of recently proposed models.

Reference	Process of generating data	Pre-processing Technique	Defect analysed	Use of ML technique	Brief summary
[245]	In Lahore, Pakistan, infrared thermal images were obtained utilizing the PV string's modules.	The utilization of a data fusion methodology for the feature extraction of RGB texturing.	Hotspots	SVM model achieved a training and testing correctness of 96.80 and 92.00 % resp.	SVM model is employed for the purpose of classifying thermal images of PV panels into 3 categories: strong, non-defective hotspots, and defective hotspots.
[246]	The I–V curve data obtained from photovoltaic (PV) modules is specifically centred on the analysis of hotspots.	Minimum-maximum averaging	Hotspots	Quantitative analysis of DT, SVM, KNN, and DC. DC showed the best detection result, whereas DT showed the worst.	Four ML models are used to diagnose early-stage hotspots in PV modules.
[240]	The design and modelling of photovoltaic systems using PSIM and MATLAB. The Agilent 34,970 datalogger is utilized for the purpose of capturing data from a 9.54 kWp Algerian grid-connected photovoltaic (PV) system.	The Canonical Artificial Bee Colony technique is utilized for the extraction of the constraints of the one diode model.	PV fault	PNN is a type of ANN that utilizes feed-forward and backpropagation algorithms for training and learning. The proposed PNN demonstrates a detection efficiency of 82.34 % and a diagnosis efficiency of 98.19 %.	The proposed PNN model has been designed for the purpose of fault identification and diagnosis inside the DC side of PV.
[247]	I–V curve data for 960 W PV array obtained from RELab JiJel university in Algeria using Prova 210 IV tracer.	The technique of dimensionality reduction via PCA and standardization.	The phenomenon of partial shade, line-to-line deterioration, and dust deposition.	The following machine learning algorithms were utilized in the study: NB, KNN, SVM, LR, DT, RF, and NN.	This investigation focuses on the application of multiple individual and combined ML for the purpose of detecting and classifying various types of PV problems.
[248]	The design of a PV with MATLAB and Simulink is being considered. The present study aims to analyse the real-time irradiance and temperature data obtained from a grid-linked PV system located in Agartala.	The technique of array capture loss was employed in the training of the machine learning algorithm.	Common flaws include Line Ground, Line, OC, arc, shading, and deterioration.	This article covers Cat Boost, LGBM, and XGBoost. The LGBM performed best, followed by CatBoost.	The PV system was modeled using Simulink and real-time data to assess and identify frequent issues.
[249]	The images obtained from photovoltaic (PV) modules that are put outside.	The technique of Gaussian blurring is commonly employed in the field of image processing.	PID and LeTID	PCA and KNN were employed in the analysis. The obtained accuracy rate with KNN was 89 %.	Modelling PCA-KNN for PID and LeTID prediction. Field-installed modules were utilized to capture EL pictures.

### 3.6 Suggestions to improve the PV system performance and its application

• One potential strategy to enhance solar energy utilization is to allocate resources towards the acquisition of high-efficiency solar panels, which have the capacity to catch a greater amount of sunlight and subsequently convert it into electrical energy.
• The integration of MPPT technology is recommended to enhance the efficiency of the PV system, particularly in situations where weather conditions fluctuate.
• It is imperative to consistently engage in the cleaning and maintenance of solar panels in order to guarantee their optimal performance by preventing the accumulation of dust and debris, which have the potential to diminish their efficiency.
• One potential strategy to address the intermittent nature of solar energy generation is the incorporation of energy storage systems, such as batteries, to store surplus energy produced during periods of peak sunlight. This stored energy can then be utilized during periods of reduced sunlight, such as cloudy days or during nighttime hours.
• This inquiry delves into the realm of innovative battery technologies, specifically focusing on lithium-ion batteries, which offer superior energy density and extended cycle life.
• The implementation of smart grid technology is crucial for the efficient management of power distribution, the optimization of supply and demand equilibrium, and the mitigation of transmission losses.
• The implementation of microgrid systems can effectively bolster energy resilience and cater to the power needs of certain localized regions, particularly in geographically isolated areas.
• The integration of novel materials, such as perovskite solar cells, into research endeavours holds promise for substantial enhancements in efficiency and reductions in production expenses.
• This study aims to investigate novel photovoltaic (PV) system configurations, such as solar trees or solar canopies, in order to optimize energy production within constrained areas.

## 4 Utilization of artificial intelligence in a desalination process operated by renewable energy

A solar still is a device that harnesses solar energy to facilitate the desalination or purification of water [12,32]. This is achieved by utilizing the sun's heat to induce evaporation of water, followed by the subsequent condensation of the resulting vapour into liquid water. The design of a solar still exhibits variability, and the integration of Artificial Intelligence (AI) and Machine Learning (ML) applications into its design is a burgeoning field with the potential to augment both efficiency and performance. This section provides a comprehensive analysis of solar still designs, examining both traditional approaches and those integrated with artificial intelligence and machine learning technologies. A conventional solar still has a shallow basin or receptacle designed to contain contaminated water, a transparent cover (often constructed from glass or plastic) to facilitate the penetration of sunlight, and a condensation surface where water vapour accumulates and undergoes the transition into liquid water. The operational principle of solar stills is based on the process of sun desalination. The water in the basin undergoes evaporation as a result of being heated by sunlight. The process involves the ascent of vapour, subsequent contact with the cover, and subsequent transformation into liquid water, which subsequently descends into a designated receptacle. The majority of solar stills are classified as passive systems, signifying their independence from external energy sources. The desalination process is exclusively dependent on solar energy.

The utilization of Machine Learning methods can be implemented to enhance the efficiency of solar still operation [250]. The algorithms possess the capability to analyse past data, weather trends, and external variables in order to make predictions regarding optimal operational timings. Additionally, these algorithms are able to alter factors such as cover inclination in order to achieve maximum efficiency. AI has the potential to automate multiple functions of the solar still, including the dynamic adjustment of the cover's position to effectively follow the sun's movement, optimising the water flow for enhanced efficiency, and effectively managing energy storage in cases where the system has such capabilities [251]. The incorporation of sensors capable of measuring factors such as humidity, temperature, and water quality enable the acquisition of real-time data for artificial intelligence (AI) systems. The information obtained can be utilized by Machine Learning models to implement real-time modifications to the operation of the solar still, hence enhancing its performance. Artificial intelligence has the potential to facilitate the solar still's ability to adjust and respond to dynamic environmental conditions. For example, the system has the capability to modify the inclination of the cover or adapt the operational parameters in response to meteorological predictions or unanticipated alterations in the surroundings. The optimization of energy storage in hybrid systems, which integrate solar stills with energy storage solutions, can be achieved through the utilization of machine learning algorithms. These algorithms facilitate the optimal utilization of energy during periods of low sunshine.

In general, the desalination process involves a variety of elements, including the allocation of a site, the optimization of a size, the selection of an operating parameter, and a variety of other difficult nonlinear problems with multiple degrees of freedom, particularly when confronted with RE systems [18]. These decisions are heavily reliant on the practical experience and pertinent standards used by researchers and industry professionals. However, in light of the progress made in science and technology, solving complex nonlinear issues solely only on an individual's expertise and mathematical equations is unattainable. However, AI excels in this area. Because of the long-term reliability and problem-solving capabilities of AI models, these models have demonstrated outstanding performance and superiority in dealing with such nonlinear data [34].

### 4.1 Different algorithms used in desalination systems

According to the published research, various kinds of AI-ML models, including ANN [252], Multilinear Regression (MLR) [253], Genetic Algorithm (GA) [254], Radial Basis Function (RBF) [255], Particle Swarm Optimization (PSO) [256], and Support Vector Machines (SVM) [257], adaptive neuro-fuzzy inference systems (ANFIS) were used to make accurate predictions and monitor water quality metrics. ANN and ANFIS algorithms are used to estimate the solar energy plant's performance and efficiency [258] however, RBF network is typically employed in modelling global solar energy [259]. The vast majority of research have monitored and evaluated the quality of surface water using neural networks (NNs). These models are able to select the most relevant information pertaining to energy systems by making use of the actual experimental data [260]. These predictive models may also help assess the productivity of new and existing solar desalination plants. Prior to conducting tests on a larger scale, these models analyse the output of solar-powered still thoroughly. As a result, both time and money are saved significantly. Such predictions make it possible to estimate whether or not the productivity of the system would be sufficient to satisfy the future needs. The WQI is the commonly used parameter for determining the performance of a water treatment plant. This parameter takes into account the individual water quality variable magnitudes of dissolved oxygen (DO), temperature, turbidity, total dissolved solids (TSS), and pH [261]. Multi-parameters are a feature of solar water desalination systems. Thus, it is essential to predict and improve operational characteristics in both the design and operational stages, including energy intake and output.

### 4.2 Overview of the utilization of AI in seawater desalination

This section analyses and summarizes the use of AI in renewable energy-based desalination of marine water. Santos et al. [262] used ANN to predict the amount of water that would be produced by two solar stills during the wintertime and summer in Nevada, United States of America. The primary purpose of this investigation was to evaluate the effectiveness of ANN models in predicting the amount of water that could be produced by a solar still using possible variants of the parameters that were provided in the introduction. Based on the input variables that were considered, it was discovered that 31–78 % of ANN predicted results were well within 10 % of the actual output.

**Fig. 9:**
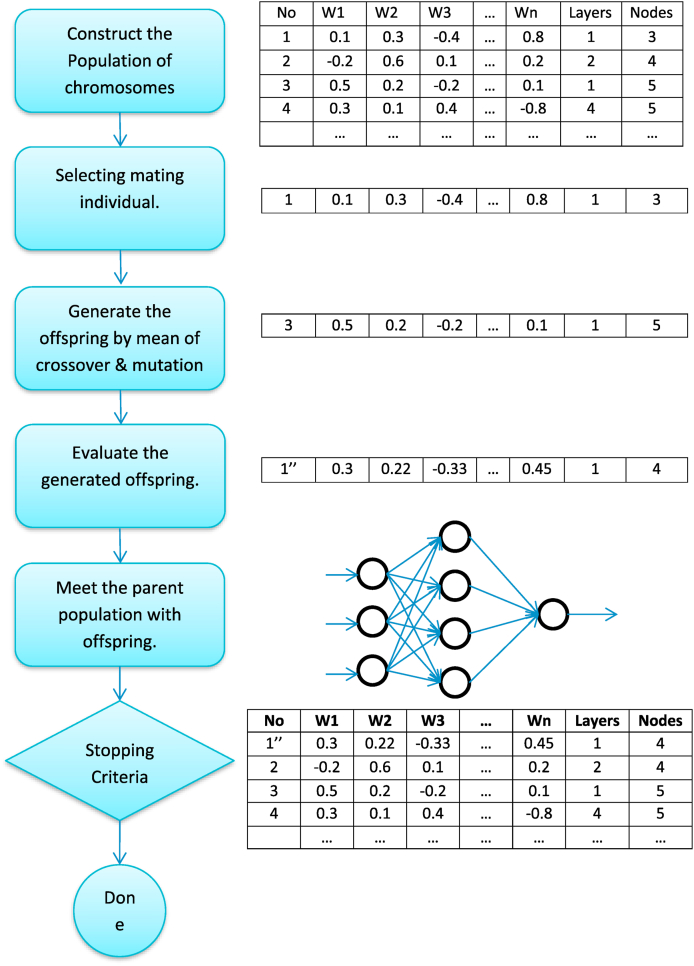
Hybrid System (GA + NN) for predicting the rate of evaporation [269] **(Redrawn and adapted with permission from ELSEV. B.V. with LIC. No.5517581259947)**.

Using an artificial neural network technique, a mathematical model was constructed to anticipate the performance of solar stills under hyper arid circumstances by Mashaly et al. [263]. Solar irradiation, Julian day, the relative humidity and temperature of the surrounding environment, the speed of the wind, the UV index, and the temperature of the feed-water were all included as input factors while production rate, recovery ration and thermal efficiency as output in the model. The authors came to the conclusion that this model had a high level of accuracy when it came to predicting the performance of solar stills. Mashaly and Alazba [264] employed ANN to forecast the efficacy of solar still production. It is an essential action to do in order to mitigate the economic threats that are connected to the desalination project. It is anticipated that the output of the solar still is a function of the following variables: the temperature, the relative humidity, the wind velocity, the radiation from the sun, the flow rates, of the water. In order to make accurate predictions of output from solar still, the authors used BP-ANN models. The authors discovered that the ANN was much more realistic than the SWR algorithm by comparing the results of their predictions with the actual findings. In AI, ANN models predict desalination performance accurately. Mashaly and Alazba [265] were employed three different learning methods for ANN that are utilized to forecast the efficacy of a solar still while it is working in a hyper-arid environment. The results demonstrated the developed model's use for making distillate output predictions with high precision. The LM algorithm performed the best across all phases of development. Furthermore, Yaci and Entchev [266] employed ANFIS to forecast the yield of sun powered still and discovered that the ANFIS methodology was great for solar stationary design. Mashaly and Alazba [267] decided to come up with several distinct sorts of input membership functions so that they could create ANFIS for the prediction of productivity of solar still. It is trained with 70 % of the data that was available, tested with 20 % of the data, and validated with the remaining 10 % of the data. It has been determined that the ANFIS model is an excellent tool for the design of solar distillation systems. ANN was the model that Abujazar et al. [268] used to anticipate the output of the stepped solar still. Authors contrasted their findings with those obtained through the use of linear and regression models. It was discovered that the model that was established provides a productivity forecast for the system that is more accurate than the linear and regression models. This made it quite evident that cascade forward ANN was able to model the solar still. In order to address the issue of roughly approximating the behaviour of evaporation rate for salty water, Salman et al. [269] suggested a novel hybrid system that combines the effectiveness of Artificial Neural Networks (NNs) and Genetic Algorithms (GA). This system is a hybrid because it combines the power of both of these types of systems. A stand-alone feed-forward neural network (NN) predicted saline water evaporation. The input layer of the neural network consists of four neurons, each corresponding to one of the four attributes utilized in the dataset. In this research, researchers performed trial-and-error experiments to determine the optimal configuration for the hidden layer of our neural network. While the specific results of these tests are not included in this paper, we found that a single hidden layer consisting of eight neurons provided the most favourable balance between accuracy and speed. The augmentation of neurons and hidden layers resulted in improved accuracy; however, it also led to an increase in the time required to attain such accuracy. The network learning rate is set at 0.2, which is a commonly recommended value for neural networks based on both empirical experimentation and existing literature. The network connection weights are randomly initialized within the range of −0.5 to 0.5. During the training process, the neural network is exposed to sample records in a random manner. Fig. 9 represents hybrid system (GA + NN) to predict evaporation rate of saline water. In this experiment, the rate of evaporation was determined for a variety of saline concentrations, water temperatures, air temperatures, and air velocities using three different experimental approaches. The result shows that generated models (GA + NN) exhibit very high degrees of accuracy.

**Fig. 10:**
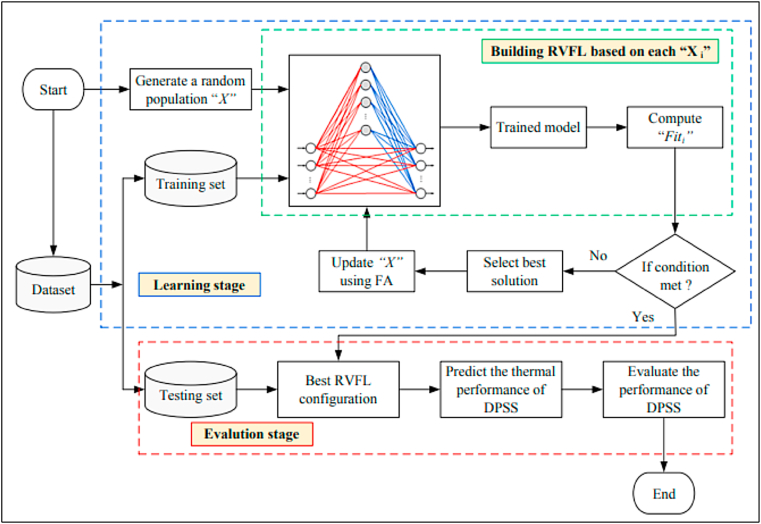
The structure of newly created method [270] **(adapted with permission from ELSEV. B.V. with LIC. No.5517480058831)**.

In order to provide accurate projections on the performance of the pyramid solar still, Sharshir et al. [270] study deals with comparison between various forecasting models, such as RVFL, SVM, and traditional ANN, along with the proposed FA-RVFL model, to forecast the characteristics of Pyramid Solar Still. The primary objective of this hyperlink is to assist the RVFL in mitigating the issue of over fitting. In the subsequent stage, the Random Vector Functional Link (RVFL) calculates the output, which represents the predicted value, by employing the subsequent equation: The equation can be expressed as Y = bw. The structure of the newly created method is depicted here in Fig. 10. Six different statistical error metrics, namely R square, RMSE, MRE, MAE, OI, and CRM, are utilized in the process of evaluating the four models that are suggested in this work. The research found that the FA-RVFL method can forecast solar still thermal performance affordably.

Chauhan et al. [271] used the feed forward BPNN to mimic the normal and redesigned solar still with a sand bed. In order to train their model, they utilized the Levenberg Marquardt method. This algorithm is a well-known hybrid algorithm that can be utilized for the purpose of converging to an optimal solution. Finally, but the authors came to the conclusion that the suggested model will be most helpful for the calculation of future distillate supply from desalination units since it avoids the obligation of extensive heat and mass transfer studies.

**Fig. 11:**
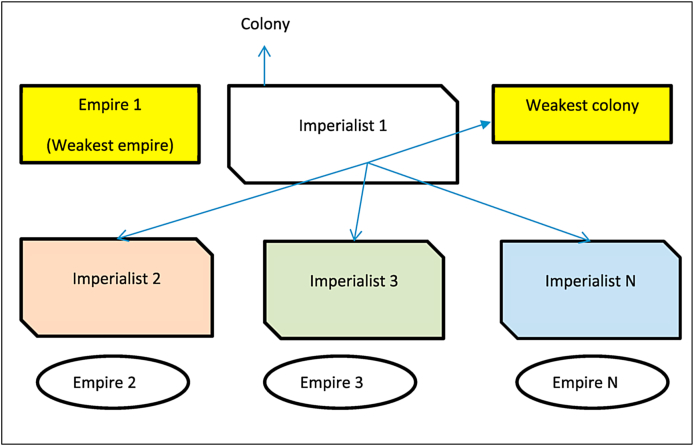
A diagrammatic representation of the ICA methodology [272] **(Redrawn and adapted with permission from ELSEV. B.V. with LIC. No 5517480702012 )**.

In the research by Nazari et al. [272], the appropriate models were developed to simultaneously forecast various parameters of solar still using ANN and ICA. Both the ANN and the ICA-enhanced ANN are trained using the empirical data that was collected. The network that has 5- hidden neurons performs the superlative out of all of the networks. According to the findings, the use of the ICA leads to a significant development in the performance of the ANN when it comes to the prediction of all three outputs represented in Fig. 11.

**Fig. 12:**
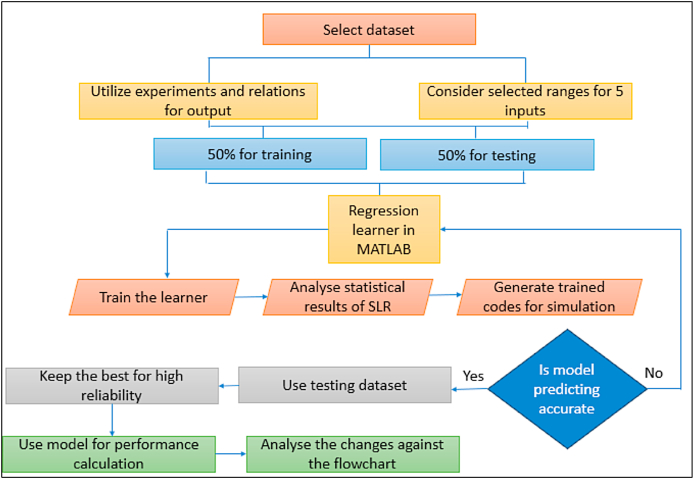
Flowchart of various actions taken during the process of developing supervised machine learning (ML) models [273] **(Adapted with permission from ELSEV. B.V. with LIC. No 5517991034624).**

Estimating the effectiveness of a solar still was accomplished by Maddah et al. [273] through the use of ML approach. Solar still walls were insulated with polystyrene. Experimental results are fed into supervised ML regressions to train models. The identical step-wise linear-regression approach was used to train polystyrene models and other insulating material datasets. Supervised ML models were developed using the procedures outlined in the flowchart shown in Fig. 12, which describes how these steps were carried out. In conclusion, these authors found that the constructed model had a high level of prediction accuracy while simultaneously having a low level of statistical error.

**Fig. 13:**
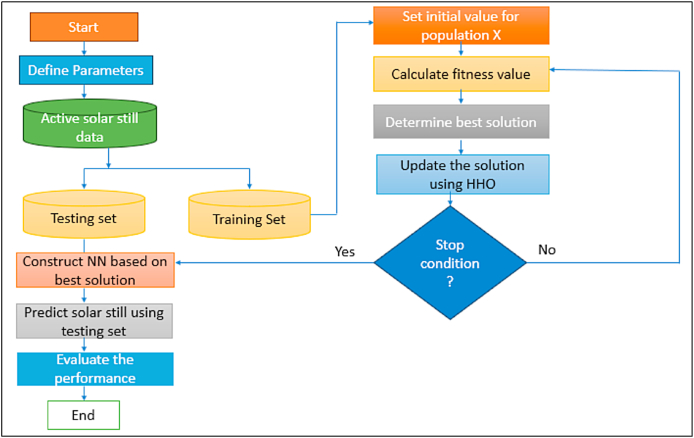
Suggested HHO–ANN approach [274] **(Adapted with permission from ELSEV. B.V. with LIC. No 5517480993818)**.

Essa et al. [274] conducted a study in which they compared the performance of three distinct desalination units—an active solar still, a condenser, and a passive solar still—using three different prediction models—the HHO-ANN, a standard ANN, and a support vector machine (SVM). The proposed HHO-ANN approach is shown in Fig. 13. In the conclusion, it was determined that out of the three models, the Harris Hawks Optimizer – artificial neural network had the most accurate prediction of the solar still yield when compared with the actual trial data. This is as a result of the ability of HHO to pick the parameters values of the ANN that produce the best results. Hence it is recommended to employ HHO-ANN to estimate the yield of the desalination system in order to avoid engaging in additional expensive and time-consuming experiments.

In order to make an accurate prediction of the tubular still, Wang et al. [275] incorporated a Bayesian optimization approach with the machine learning techniques. The ANN and RF algorithms were utilized as ML strategies in this particular piece of work. Using ML approaches, the results indicated that it is possible to make predictions for hourly production that are closer to genuine experimental data. In addition, the use of BOA rather than RF can lead to a significant improvement in ANN's overall performance. This suggests that RF is not as susceptible to hyper parameters as ANN. In other words, the RF model is more reliable than the ANN model. Despite the fact that ANN has successfully forecasted on a variety of large datasets, the prediction performance of TSS on the present dataset was improved by RF. It is advised that RF be used as a reliable method for forecasting the productivity of TSS due to its high level of accuracy and high level of durability.

**Fig. 14:**
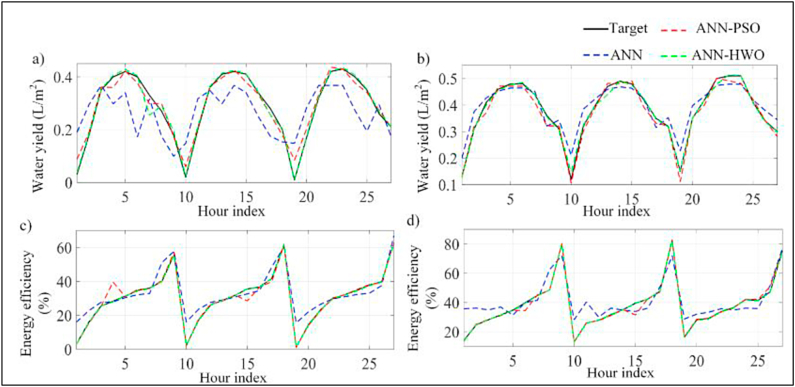
The measured and predicted using the three models for: A) Water yield of CTSS [276]; B) water yield of MTSS [276]; C) energy efficiency of CTSS [276]; D) energy efficiency of MT*S*S [276].

The researchers Moustafa et al. [276] constructed a model using artificial intelligence that was fine-tuned in order to anticipate the still's efficacy. A standard artificial neural network model has been fine-tuned humpback-whale-optimizer (HWO). Comparisons are made between the accuracy of predictions made by the produced model, and those made by a ANN and an optimized model created with PSO. The data is broken down into two categories: the training group, which accounts for 80 % of the total, and the test group, which accounts for 20 %. Fig. 14 compare expected and measured water yield and efficacy. The single ANN model has the weakest agreement with experimental results (in blue). This chart shows the role of metaheuristic optimizers, since ANN-HWO and ANN-PSO forecast better experimental results than ANN.

**Fig. 15:**
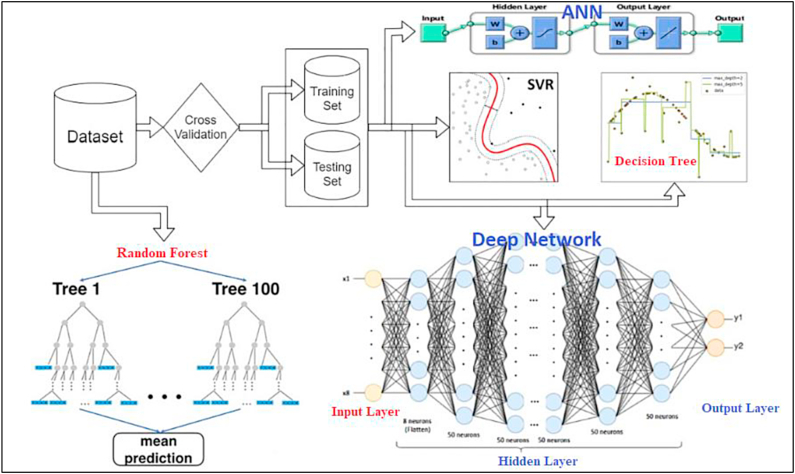
Diagrammatic representation of the process of training [19].

In the research carried out by Sharshir et al. [19], a assessment of four different algorithms is made in order to generate a prediction model of the performance of tubular still. Based on experimental data, the SVR, DTR, NN, and DNN are the methodologies that were created and compared. To begin, all of the data from the dataset was run through a random forest regressor to determine the relative weight of each input feature in terms of its percentage importance. Fig. 15 shows the Diagrammatic representation of the process of training. The results show that the prediction performance for the SVR was the worst.

**Fig. 16:**
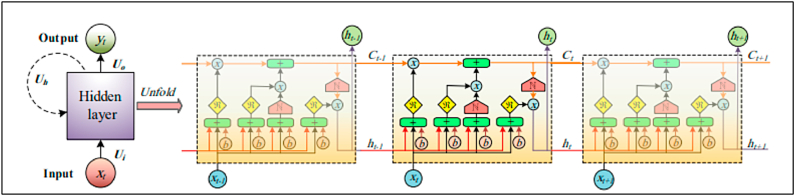
Typical structure of LSTM model [277] **(Adapted with permission from ELSEV. B.V. with LIC. No 5517490433132)**.

Elsheikh et al. [277] used a long short-term memory NN to predict the amount of freshwater that would be generated by a stepped still. When contrasted with the conventional feed-forward ANN, the capability of this neural network to remember patterns for an extended period of time stands out as the most significant advantage offered by it. This function is made possible by the highly developed structure that is associated to feedback linkages. This network has the capability to handle sequence predicting, which is an important feature. Fig. 16 shows the typical structure of LSTM model. Several distinct statistical measures were utilized to evaluate and compare the accuracy of the suggested model's predictions. The R^2^, RMSE, MAE, EC, and OI values for the stepped solar still are 0.9976, 0.0021, 0.0018, 0.9973, and 0.9897.

In order to demonstrate the dependability of solar distillation, a parametric analysis was carried out using a model that was based on artificial neural networks which is developed by Labbadlia et al. [278] this study was performed on two different types of stills. The usage of ANN approach has an impact on the analysis of thermal parameters of traditional solar stills and capillary film solar stills. This research was carried out with the purpose of developing models of artificial neural networks that may be utilized to anticipate the daily output of a solar distiller. The results inferred that a capillary film solar still when utilizing ANN for the prediction of daily production with a correlation coefficient of approximately 0.99.

The author Maddah [279] constructed accurate supervised predictive machine learning models for the performance predictions in a double-slope still based on the experimental data found in the relevant body of literature. The previous findings (inputs/outputs) from various designed passive and/or active solar stills that were used to treat brackish water or wastewater with 45 % TDS were utilized to generate training datasets. These stills were used to treat brackish water or wastewater. The fact that the regression models (FGSVM, EBoT, and SEGPR) achieved the lowest RMSE demonstrates their reliability to accurately estimate the amounts of distillate in double-slope designs. The potential of the model to accurately forecast the concert of other desalination systems was demonstrated by the extremely high accuracy of the SEGPR trained model with (R^2^ = 1) and the extremely low RMSE (<8)

The model that was proposed by al-sulttani et al. [280] is employed for the determination of the best values for the unidentified constant (C) and the exponent (n) for the Nusselt number expression that was used to formulate the equation for the estimation of the hourly yield of a solar still. This was accomplished by finding a solution to an optimization problem by using PSO model. During this process, the optimal yields were found by estimating the optimal values for the unknown C and n parameters, which led to the successful completion of the optimization problem. This approach, which is employed for the first time ever in this study to create a yield prediction model and it, avoids the typical trial-and-error approach to estimating unknown coefficients. Based on the findings, the PSO algorithm appears to be a useful tool for solving solar distillation-related problems; specifically, it yields an optimum solution for estimating HYSS from a number of input variables.

Zayed et al. [281] developed two algorithms with diverse kernels to forecast the water output of two sun distillers of varied configurations. The RVM and LS-SVM are two different techniques to machine learning. The output of two different solar stills can be predicted with the use of the RVM and LSSVM models that have been proposed. The RVM is a ML model that incorporates probabilistic principles, where the weights are utilized as hyper priors. This study employs the RVM for the purpose of forecast applications. The idea of structure in the context of Reliability, Availability, and Maintainability (RAM) prediction using the Reliability, Availability, and Maintainability RVM model encompasses a series of four distinct processes. The input feature vector for the RVM consists of three features and is represented as follows: The variable X is a vector consisting of three elements, denoted as x1, x2, and x3. The RVM model is structured as a three-layer network, with the first layer comprising the features.

**Fig. 17:**
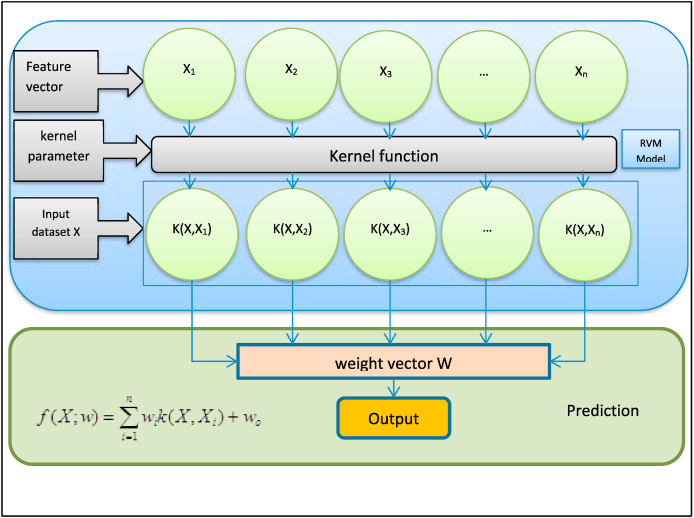
Flowchart of RVM model [281] **(Adapted with permission from ELSEV. B.V. with LIC. No 5517530853157)**.

Fig. 17 is illustration that demonstrates the RVM structural idea. During the course of the simulation, a number of distinct kernel functions, including Laplace, Gaussian (G), linear, spline, and radial basis function (RBF), were incorporated into both models in an effort to determine which kernel function is the most effective in terms of maximizing the accuracy of the model. The results of the simulations demonstrate that the Gaussian function and the radial basis function are the most suitable Kernel functions to use for the RVM and LSSVM algorithms, respectively.

In a study that was carried out by Nazari et al. [282], four reliable ML approaches were used to assess the efficacy of solar stills. In the context of this research, the impacts of various input parameters were explored. Through a process of training and testing, different parameters were predicted using a variety of ML methods. Further, mathematical equations had to be extracted from experimental results and used as a basis. In this manner, statistical measurements were utilized in order to assess accurate equations during both the preparation and testing stages. In addition, the outcomes of ML models were associated with the research that has been done in the past in terms of correctness and accuracy. The outcomes of ML models indicated that the EPR technique yielded comparatively better performance in the prediction of energy.

As one of the most common approaches to machine learning, artificial neural networks (ANN) are developed in a variety of forms, and then those forms are compared with one another to determine which one performs the best at predicting the distillate and the temperature of the water. These are two of the most important performance criteria of the system designed by Sohani et al. [283]. Analysis is conducted on the FF, BP, and RBF varieties of ANN. The findings indicate that the FF and RBF types of ANN structures are the most effective for estimating the distillate and the temperature of water.

In the research carried out by Kandeal et al. [284], a total of four distinct ML approaches-namely; ANN, RF, SVR, and L-SVR were utilized in order to make an accurate prediction regarding the concert of the still when combined with the Nano fluid. The coefficient for the RF model, the ANN model, linear SVR, and the SVR model, respectively, were 0.997, 0.986, 0.956, and 0.99. Among the four ML models, the RF provides better prediction, with the maximum coefficient of determination and the least absolute percentage error.

Bahiraei et al. [285] has created an effective model for estimating the efficacy of a still. In this model PSO is used in conjunction with an ANFIS to further improve the performance of an ANN. The best predictions found the ANN model having three hidden neurons and the ANFIS model with nine clusters. By comparing these models with one another, the performances of PSO-based ensemble models demonstrate that the PSO-ANFIS is superior to the PSO-ANN. When applied to the training set, the PSO-ANFIS model achieves an R^2^ value of 0.9884.

**Fig. 18:**
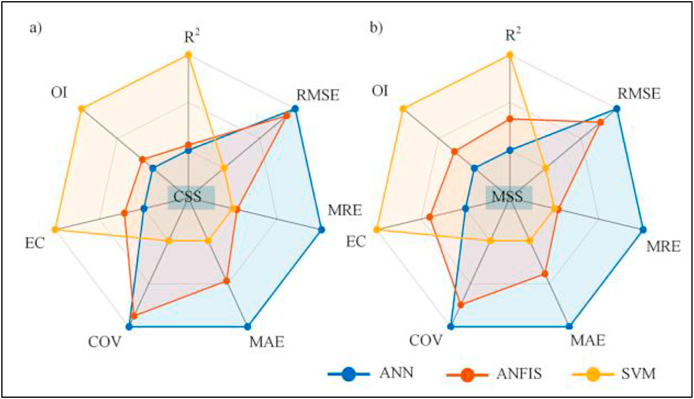
Spider plots comprised of a number of different statistical measures A) conventional [286] B) modified solar still [286] **(Adapted with permission from ELSEV. B.V. with LIC. No. 5517531385260)**.

Elsheikh et al. [286] carried out a research in which authors forecasted the yield of stills by utilizing different ML approaches. These techniques included SVM, ANN, and ANFI system. The research specially deals with two stills which include a conventional solar still and an enhanced model that featured a bilayered structure. The spider graphs of several numerical metrics used for evaluation of ML approaches for the two solar stills are illustrated in Fig. 18. This figure demonstrates that, according to a variety of statistical criteria, SVM performs better than former algorithms for analysing both traditional and adapted stills.

**Fig. 19:**
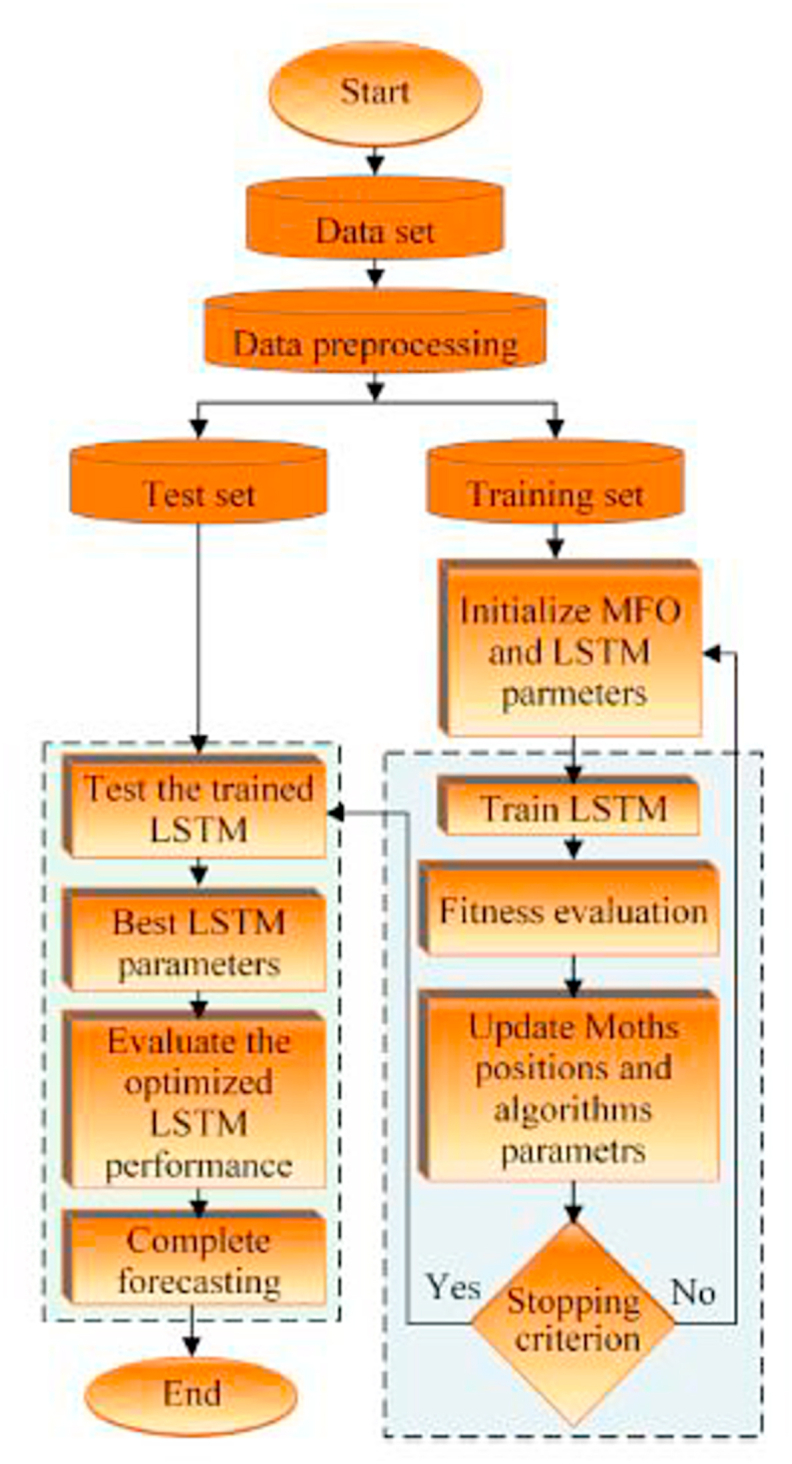
The operational steps of the LSTM-MFO model [287].

Elsheikh et al. [287] devised a model using AI to predict the volume of water that could be created from modified distiller. The model is made up of LSTM that has been optimized using MFO. The concert of the model was analysed and contrasted with that of a LSTM model. The cellular state is regarded as the central component of the entity. Information is moved via a conveyor belt across the memory cell. Cell state is regulated by input, forgetting, and output gates. The sigmoid function has a limited output between 0 and 1. Zero units mean no information transmission. The unit can pass all information if its value is one. The forget gate decides which non-essential cell information to omit. The processes for carrying out the LSTM-MFO model that has been developed are laid out in Fig. 19. After undergoing pre-processing, the data used for training are separated into two distinct groups: the test set and the training set.

**Fig. 20:**
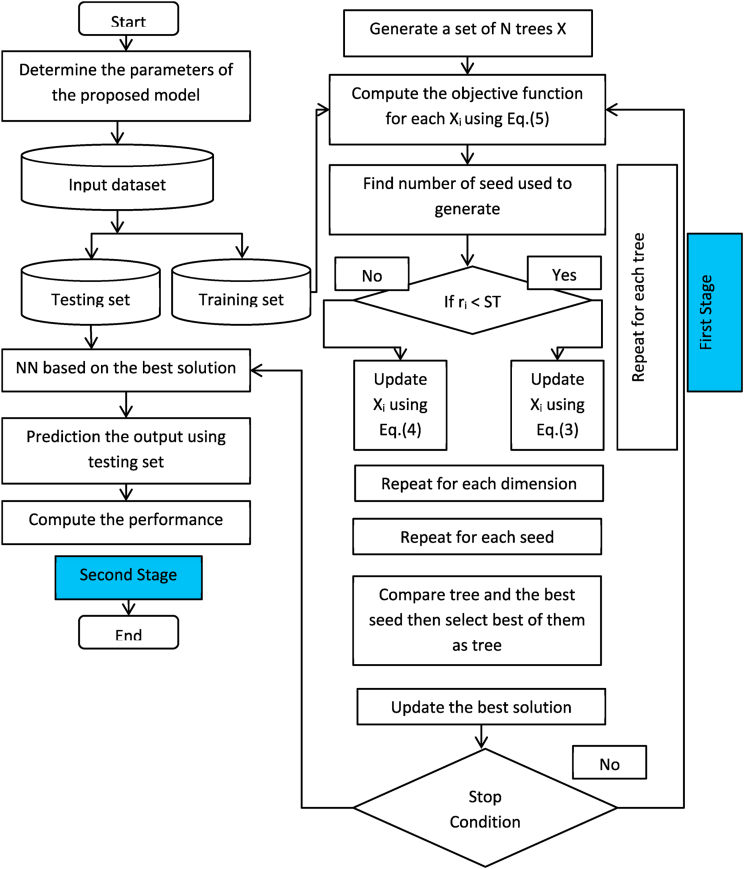
Organizing framework of suggested technique [288] **(Adapted with permission from ELSEV. B.V. with LIC. No 5517591114478)**.

A modified ANN model created on the tree–seed algorithm is presented by Sharshir et al. [288] in order to estimate the water volume of conventional and wick stills. The usefulness of this method is contingent upon enhancing the functionality of the ANN by locating the optimum weights for the neurons through the utilization of the TSA. The initial phase involves the reception of the input data, which is subsequently divided into training and test sets by a random allocation process. Subsequently, a collection of solutions X is generated that correspond to the weights of the NN. In the second stage, the testing set is employed to evaluate the trained NN using the optimal solution X superlative. Next, the productivity is evaluated, and the output is assessed. The evaluation of this process is conducted by contrasting it with a conventional ANN. The procedure of defining the constraints of TSA is typically conducted through a trial-error approach. It is observed that a population size of 25 is commonly selected. Fig. 20 illustrates the organizing framework of the technique that is being suggested. The suggested approach (ANN-TSA) will be perceived in 2 steps. Initial stage will seek to recognize the weights that will result in the lowest RMSE value when comparing the output to its projected values. In the meantime, the purpose of the second step is to measure the level of quality of ANN. The study's findings indicate that ANN-TSA has the potential to be an extremely efficient for forecasting productivity.

### 4.3 Suggestions to improve the solar desalination performance and its application

• To improve the total efficiency of freshwater production, try more sophisticated condensation methods like multi-stage solar stills.
• To optimize energy intake, it is recommended to incorporate both passive and active solar tracking devices, which will ensure that the still remains oriented with the sun at all times.
• Utilize advanced materials such as hydrogels or super-hydrophobic coatings on the solar still's surface to optimize heat absorption and augment the condensation mechanism.
• Examine materials with nanostructures to improve condensation rates and sunlight absorption.
• One potential approach to enhance evaporation rates in a still is to introduce reflectors or concentrators, which can effectively redirect a greater amount of sunlight onto the surface of the still.

## 5 Discussion on unresolved issues from current review article

The rapidly changing environment of innovation is illuminated by the new energy scenarios that are emerging around the world. This covers the development of new social innovations, commercial strategies, and policy as well as new energy technology. AI will drive every aspect of business, policies, and energy innovations including repairs, unit sizing, automated contracting, logistics optimization, and others. The development of ML models and DL models, and novel search strategies into a general knowledge of the globe, are the primary accomplishments in AI over the previous seventy centuries. The application of AI, which is a very vital tool, has the potential to drastically alter the energy market if it is done efficiently. These tools are only as effective as their training and information sources. Many procedures, especially energy industry safety-critical ones, may have a black box component.

**Fig. 21:**
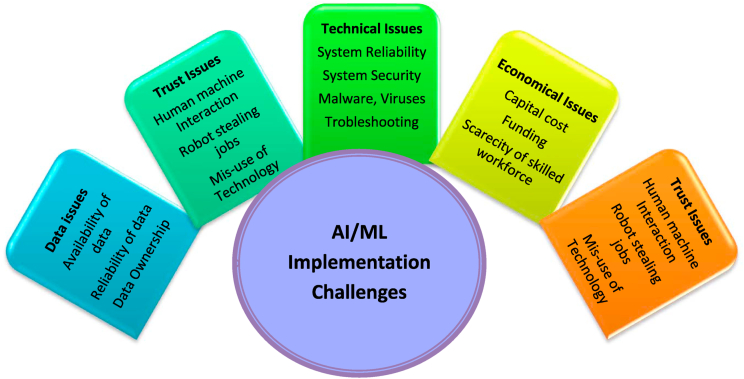
Different Challenges in an implementation of AI-ML [289].

The application of AI presents a great chance to overhaul both the modern energy sector and modern energy systems, but it also presents a number of challenges represented in Fig. 21. Yet, there is still some obstacle that prevents the widespread use of AI from progressing further. Some examples of hurdles are those pertaining to the effective tuning of network hypermeters, problems with data quality, a dearth of skilled experts and data science abilities, problems with the technological infrastructure, compliance issues, concerns about legal security, and so on. There are a lot of issues to overcome in terms of technology, the lack of openness of AI approaches and the challenges the AI system has interpreting unstructured data.

## 6 Recommendations from the results of current review article

The past results give us reason to recommend that the power and energy society increase their efforts to engage AI approaches in fault identification and categorization. These efforts should be increased because the prior results gave us reason to do so. Moreover, the deployment of AI in energy systems might prevent any breakdowns, which would result in a significant increase in the amount of electricity generated. In addition, utilizing AI-based approaches can make the process of maintenance much simpler. The authors suggest giving data-gathering operations, which can speed up the implementation of AI approaches in the power and energy sectors, more attention in order to improve their effectiveness. The efforts of the government ought to also be focused on the same aim. It is strongly suggested that additional investments be made in the research and development of various monitoring strategies. These methods could be helpful in acquiring the data with high levels of accuracy and in a very short amount of time. It's important to create a machine learning approach that includes measurement and precaution and meets international standards like IEC, NEC, and UL to get reliable results that account for outside influences and measuring device signal variability. The Internet of Things (IoT) is highly suggested for use in the development of FDD-based smart monitoring systems and in the remote sensing of PV plants. In addition, it is strongly recommended to use a strategic strategy in order to quickly isolate the plants and provide them with immediate protection [[290], [291]].

## 7 Conclusions

Incorporation of AI and ML in the dynamic field of solar technology is a significant achievement, marking the beginning of a new era characterized by innovation and enhanced productivity. This review paper thoroughly examines the diverse uses and significant influence of AI and ML in the photovoltaic systems and solar desalination, providing insight into the revolutionary capabilities of these technologies.

• Recent study indicates that AI and ML algorithms have surpassed conventional limitations, enhancing different aspects of PV systems. AI-driven technologies have transformed the utilization of solar energy by enabling predictive maintenance, defect detection, real-time monitoring, and performance optimization.
• These advancements have resulted in increased efficiency, decreased expenses, and improved sustainability. Moreover, the ML algorithm possesses the ability to adapt photovoltaic systems effectively to react with fluctuating environmental conditions, hence optimising energy production even under uncertain circumstances.
• Furthermore, the integration of AI and ML with solar technology has not only increased operational efficiency, but also expedited the rate of innovation. Researchers and industry experts can now investigate new materials, designs, and production processes using intelligent algorithms that evaluate large datasets and make predictions.
• This study summarizes the major factors in developing and operating renewable energy-powered desalination methods. These include choosing a location, energy estimation, desalination method selection, and system efficiency enhancement. These four issues can be solved individually with AI.
• The two most frequently employed intelligent algorithms in the context of renewable driven desalination are Artificial Neural Networks (ANN) and Genetic Algorithms (GA). ANNs have proven to be valuable tools in the prediction of desalination processes. Additionally, GAs is commonly employed in the optimization process, given their advantageous characteristics.
• By utilizing artificial intelligence technologies, freshwater productivity can increase by 10 % and efficiency can be improved.

## 8 Future directions

The readers are provided with future scope in order to help them form a compelling view on the emerging developments that will occur in this field, including themes that should be developed further.

• The implementation of applications that make use of AI and ML approaches has to be made simpler, more effective, and more cost-effective, and there are a number of different areas in which additional study is required.
• There is little experimental integration of ML, DL, and IoT technologies online, and they should run in actual time to prove their efficacy.
• Big data and advanced deep learning algorithms make fault prediction possible, making this a promising field for the future.

## CRediT authorship contribution statement

**Laxmikant D. Jathar:** Writing – review & editing, Software, Visualization, Writing – original draft, Conceptualization. **Keval Nikam:** Writing – review & editing, Writing – original draft, Conceptualization. **Umesh V. Awasarmol:** Resources, Writing – review & editing, Writing – original draft, Conceptualization. **Raviraj Gurav:** Writing – review & editing, Writing – original draft, Conceptualization. **Jitendra D. Patil:** Writing – review & editing, Methodology, Conceptualization. **Kiran Shahapurkar:** Writing – review & editing, Resources, Conceptualization. **Manzoore Elahi M. Soudagar:** Project administration, Writing – review & editing, Supervision, Writing – original draft, Conceptualization. **T.M. Yunus Khan:** Writing – review & editing, Funding acquisition, Conceptualization. **M.A. Kalam:** Writing – review & editing, Formal analysis. **Anna Hnydiuk-Stefan:** Writing – review & editing, Resources, Conceptualization. **Ali Etem Gürel:** Writing – review & editing, Conceptualization. **Anh Tuan Hoang:** Writing – review & editing, Supervision. **Ümit Ağbulut:** Writing – review & editing, Project administration, Conceptualization.

## Declaration of competing interest

We, the authors of this manuscript do not have any conflict of interest including any financial, personal or other relationships with other people or organizations within three years of beginning the submitted work that could inappropriately influence, or be perceived to influence, our work.
